# Full Copper Resistance in Cupriavidus metallidurans Requires the Interplay of Many Resistance Systems

**DOI:** 10.1128/aem.00567-23

**Published:** 2023-05-16

**Authors:** Niklas Hirth, Michelle-Sophie Gerlach, Nicole Wiesemann, Martin Herzberg, Cornelia Große, Dietrich H. Nies

**Affiliations:** a Molecular Microbiology, Martin-Luther-University Halle-Wittenberg, Halle, Germany; Colorado School of Mines

**Keywords:** Cupriavidus metallidurans, P-type ATPases, copper resistance, multicopper oxidases

## Abstract

The metal-resistant bacterium Cupriavidus metallidurans uses its copper resistance components to survive the synergistic toxicity of copper ions and gold complexes in auriferous soils. The *cup*, *cop*, *cus*, and *gig* determinants encode as central component the Cu(I)-exporting P_IB1_-type ATPase CupA, the periplasmic Cu(I)-oxidase CopA, the transenvelope efflux system CusCBA, and the Gig system with unknown function, respectively. The interplay of these systems with each other and with glutathione (GSH) was analyzed. Copper resistance in single and multiple mutants up to the quintuple mutant was characterized in dose-response curves, Live/Dead-staining, and atomic copper and glutathione content of the cells. The regulation of the *cus* and *gig* determinants was studied using reporter gene fusions and in case of *gig* also RT-PCR studies, which verified the operon structure of *gigPABT*. All five systems contributed to copper resistance in the order of importance: Cup, Cop, Cus, GSH, and Gig. Only Cup was able to increase copper resistance of the Δ*cop Δcup Δcus Δgig ΔgshA* quintuple mutant but the other systems were required to increase copper resistance of the Δ*cop Δcus Δgig ΔgshA* quadruple mutant to the parent level. Removal of the Cop system resulted in a clear decrease of copper resistance in most strain backgrounds. Cus cooperated with and partially substituted Cop. Gig and GSH cooperated with Cop, Cus, and Cup. Copper resistance is thus the result of an interplay of many systems.

**IMPORTANCE** The ability of bacteria to maintain homeostasis of the essential-but-toxic “Janus”-faced element copper is important for their survival in many natural environments but also in case of pathogenic bacteria in their respective host. The most important contributors to copper homeostasis have been identified in the last decades and comprise P_IB1_-type ATPases, periplasmic copper- and oxygen-dependent copper oxidases, transenvelope efflux systems, and glutathione; however, it is not known how all these players interact. This publication investigates this interplay and describes copper homeostasis as a trait emerging from a network of interacting resistance systems.

## INTRODUCTION

**FIG 1:**
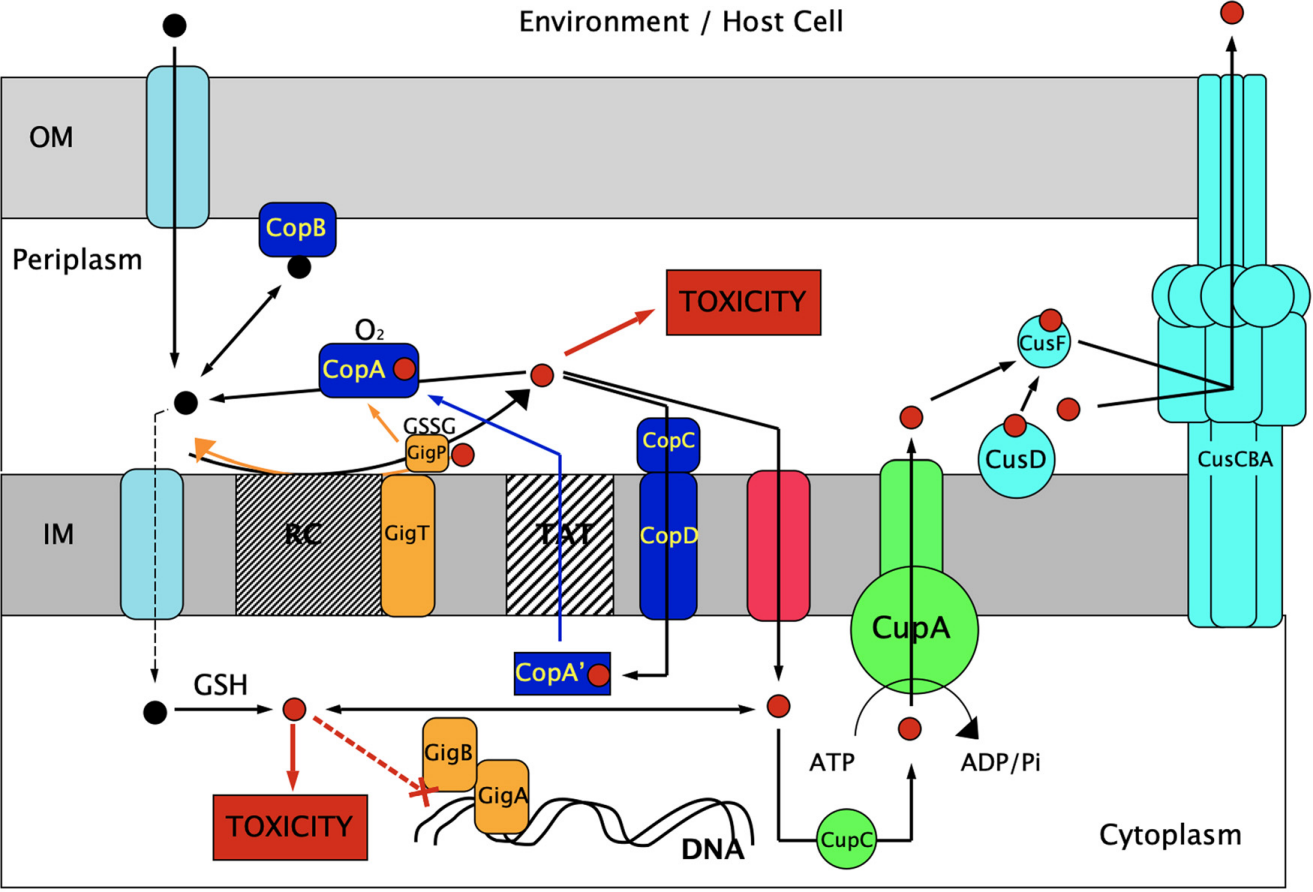
Model for copper homeostasis in *C. metallidurans*. In the plasmid-free *C. metallidurans* strain AE104, which does not possess the pMOL30-encoded *cop* and *sil* determinants (3), Cu(II) (black dots) is imported into the periplasm of this Gram-negative bacterium by outer membrane (OM) porins and further on with a low rate into the cytoplasm as unspecific substrate of metal cation import systems such as ZupT (light blue) (48). Periplasmic Cu(II) ions are reduced upon contact with the respiratory chain (RC) to Cu(I) (red dots) (45) and cytoplasmic Cu(II) ions via glutathione (GSH) (28), which leads to toxic effects in both compartments. Periplasmic Cu(I) is a much better substrate for import and is subsequently imported into the cytoplasm at a higher rate than Cu(II) by unspecific import (red system) (9, 10). The central component of the Cop system (dark blue) is the copper- and oxygen-dependent oxidase CopA, which oxidizes Cu(I) back to Cu(II) (69, 70, 126, 127), decreasing copper import into the cytoplasm. Cu(II) is sequestered by CopB, which is attached to the OM (75). CopA is exported to the periplasm by the twin-arginine transport system (TAT) in a partially folded, probably copper-loaded form CopA’ (74) with CopCD-mediated import or GSH-mediated reduction of Cu(II) providing the required copper ions (72, 73). The Cup system (green) is centered around the P_IB1_-type, Cu(I)-exporting ATPase CupA, which may receive Cu(I) from the cytoplasmic copper chaperone CupC (128–130). The Cus system (green-blue) exports periplasmic Cu(I) from the periplasm to the outside by the transmembrane efflux system CusCBA, which may receive Cu(I) from the periplasmic copper chaperone CusF (19). CusF may have previously accepted Cu(I) from CupA or the IM-attached protein CusD. GigBA (orange) may protect the DNA or DNA-operating enzymes against copper toxicity. GigT in combination with GigP and oxidized glutathione (GSSG) may oxidize periplasmic Cu(I) to Cu(II) by feeding the electron into the quinol pool and GigP may additionally assist in maturation of CopA.

The betaproteobacterium Cupriavidus metallidurans survives in environments rich in transition metals (1–3). The necessary metal resistance determinants are located on the bacterial chromosome, a chromid and two plasmids (4). *C. metallidurans* also occurs in biofilms of bacterio-formed gold (5). In auriferous soils, gold complexes are rapidly accumulated within the cell but later precipitated as gold nanoparticles in the periplasm (6). Upon contact with gold complexes, defense systems against oxidative stress and copper resistance systems (Fig. 1 and Fig. S1 in the supplemental material) are upregulated (7), for instance, the yet uncharacterized *gig* genes (gold induced genes, Fig. S1A). Auriferous soils usually contain an elevated copper content. Copper ions and gold complexes exert synergistic toxicity because cytoplasmic gold compounds inhibit the P_IB1_-type ATPase CupA, which is responsible for removal of surplus cytoplasmic Cu(I) ions (Fig. 1, Fig. S1D) (8). To prevent this combined toxic effect, *C. metallidurans* induces the *cop* determinant. The periplasmic Cu(I)/Au(I) oxidase CopA (Fig. 1, Fig. S1E) oxidizes both compounds back to Au(III) and Cu(II), which are less toxic than the respective monovalent ions (6, 7, 9, 10). Au(III) complexes can be subsequently reduced to metallic Au(0) in the periplasm, leading to direct formation of gold nanoparticles in the periplasm without using the toxic Au(I) state as an intermediate (11).

This scenario highlights that gold transformation and resistance is interwoven with copper resistance and homeostasis in *C. metallidurans*. Gold resistance and transformation of *C. metallidurans* CH34 wild type and its plasmid-free derivative strain AE104 is similar (7), so that plasmid pMOL30 with its large copper resistance gene region (12, 13) does not need to be considered to understand the copper-gold interaction network. *C. metallidurans* possesses, in addition to CupA, three other P_IB1_-type, copper exporting ATPases, pMOL30-encoded CopF and chromosome-encoded CtpA1 and RdxI. CtpA1 and RdxI are “anabolic” exporters that supply copper ions to periplasmic copper binding sites of cuproproteins (14). Their genes are not upregulated by gold compounds in contrast to *cupA* (7). The contribution of these proteins to copper resistance has already been characterized (8).

Resistance-nodulation-cell division family of proteins (RND)-driven transenvelope efflux systems (15) such as CusCBA from E. coli export Cu(I) and Ag(I) from the periplasm to the outside (16–19), so that the orthologue CusCBA from *C. metallidurans* (Fig. 1, Fig. S1C) may also export Au(I), reminiscent to the RND-type GesCBA transport system in Salmonella (20, 21). The thiol-containing tripeptide glutathione (GSH; Fig. 1, Fig. S1B) is involved in copper resistance of Escherichia coli (10, 22) and present in many bacteria (23). It is synthesized by the gamma-glutamylcysteine synthetase GshA and the glutathione synthetase GshB from glutamate, cysteine, and glycine (24). If copper is imported into the cytoplasm as Cu(II), the ion should be immediately reduced by GSH to Cu(I) and GS-SG, and subsequently bound to GSH (25–28), until Cu(I) can be sequestered by copper chaperones such as CupC for further delivery to a copper-exporting P-type ATPase (29–32).

A first step into a deeper insight into the synergistic toxicity of copper and gold compounds, as well as into the synergetic gold-copper detoxification, would be an analysis of the network of copper resistance determinants in the plasmid-free *C. metallidurans* strain AE104 (Fig. 1). In this publication, we investigate the interplay of systems centered around the P_IB1_-type ATPase CupA, the periplasmic copper oxidase CopA, the RND efflux system CusCBA, or the unknown Gig components, respectively, with each other in the absence and presence of glutathione.

## RESULTS

### Contribution of Cup, Cop, Cus, Gig, and glutathione to copper resistance in *C. metallidurans*.

Single, double, triple, quadruple mutants, and the quintuple mutant in the *cupC/AR*, *copA_2_B_2_C_2_D_2_*, *cusDCBAF*, *gigTBAP*, and *gshA* gene regions (further referred to a Δ*cup*, *Δcop*, *Δcus*, *Δgig*; Fig. S1) were constructed in the plasmid-free *C. metallidurans* strain AE104. While a mutant carrying a marker-free deletion of *gshA* could be obtained in the parent strain background, it was neither possible to construct marker-free deletions of *gshA* in the strains with deletions in the copper resistance determinants nor to delete copper resistance determinants in the Δ*gshA* mutant. Consequently, *gshA* was interrupted in all these strains, which also should lead to a decreased expression of *gshB* due to a polar effect (Fig. S1B). Tables 1, 2, and 3 show only the results from the Δ*gshA* mutants with the interrupted gene, and Table 4 included the comparison with the marker-free Δ*gshA* deletion in the parent strain AE104.

**TABLE 1:** Copper resistance of strains with and without disruption of Δ*gshA*^*a*^

Bacterial strain	IC_50_ (μM)	Bacterial strain	IC_50_ (μM)	Q	D
*Δcus*	743 ± 62	*Δcus ΔgshA*	612 ± 32	1.21	1.40
AE104	615 ± 68	*ΔgshA*	450 ± 6	1.37	2.22
*Δcus Δgig*	597 ± 26	*Δcus Δgig ΔgshA*	472 ± 49	1.26	1.68
*Δgig*	580 ± 30	*Δgig ΔgshA*	458 ± 43	1.26	1.67
*Δcop Δgig*	492 ± 50	*Δcop Δgig ΔgshA*	110 ± 9	4.46	6.40
*Δcop*	389 ± 43	*Δcop ΔgshA*	161 ± 24	2.41	3.37
*Δcop Δcus*	197 ± 21	*Δcop Δcus ΔgshA*	26.4 ± 2.9	7.45	7.18
*Δcop Δcus Δgig*	158 ± 16	*Δcop Δcus Δgig ΔgshA*	29.0 ± 4.4	5.44	6.41
*Δcup*	13.5 ± 1.2	*Δcup ΔgshA*	13.2 ± 2.5	*1.02*	0.07
*Δcup Δgig*	11.0 ± 2.1	*Δcup Δgig ΔgshA*	6.22 ± 1.03	1.77	1.52
*Δcup Δcus*	4.25 ± 0.89	*Δcup Δcus ΔgshA*	3.83 ± 1.37	*1.11*	0.19
*Δcop Δcup*	3.41 ± 0.62	*Δcop Δcup ΔgshA*	0.42 ± 0.10	8.12	4.15
*Δcup Δcus Δgig*	2.22 ± 0.14	*Δcup Δcus Δgig ΔgshA*	0.51 ± 0.12	4.35	6.58
*Δcop Δcup Δcus*	0.81 ± 0.23	*Δcop Δcup Δcus ΔgshA*	0.43 ± 0.09	1.88	1.19
*Δcop Δcup Δcus Δgig*	0.47 ± 0.10	*Δcop Δcup Δcus Δgig ΔgshA*	0.44 ± 0.16	*1.07*	0.12
*Δcop Δcup Δgig*	0.43 ± 0.21	*Δcop Δcup Δgig ΔgshA*	0.46 ± 0.07	*0.93*	0.11

a Copper resistance was determined in 96-well plates at 30°C. These data are sorted in decreasing order of the IC_50_ values of the *gshA*^+^ strains. The Q values give the IC_50_ ratios plus and minus *gshA*. D, respective distance value. Q values in italics indicate no difference between the IC_50_ value (D < 1); bold-faced values indicate Q > 2. Table S1 compares the effect of other deletions. The D value is used as an easy-to-handle statistical value and is the ratio of the absolute difference of the mean value of two data sets, divided by the sum of the respective deviations. At *n* ≥ 3, D > 1 means a significance of 95% (33, 34). D > 1 means that the deviation bars of two mean values do not overlap or touch.

**TABLE 2:** Percentage of dead cells in cultures of *C. metallidurans* mutant cells^*a*^

Bacterial strain	% dead cells			
Glutathione	Present (GSH^+^)		Absent (Δ*gshA*)	
Added Cu(II)	No	At IC_50_	No	At IC_50_
*Δcus*	2.81 ± 2.72	***15.9 ± 16.7***	1.32 ± 0.58	**7.04 ± 4.36**
AE104	4.93 ± 2.40	***9.12 ± 5.98***	2.81 ± 1.76	***5.54 ± 3.23***
*Δcus Δgig*	3.41 ± 3.88	***18.2 ± 26.4***	2.24 ± 0.44	3.64 ± 2.07
*Δgig*	3.45 ± 0.71	5.73 ± 2.68	2.54 ± 1.91	***4.10 ± 2.38***
*Δcop Δgig*	2.51 ± 2.30	***10.1 ± 10.1***	4.50 ± 0.39	**30.7 ± 10.0**
*Δcop*	2.90 ± 1.58	***9.67 ± 5.66***	2.74 ± 3.04	***4.69 ± 3.28***
*Δcop Δcus*	3.57 ± 2.11	**27.3 ± 17.1**	2.75 ± 1.89	**13.7 ± 5.6**
*Δcop Δcus Δgig*	5.51 ± 3.13	***17.1 ± 6.1***	6.54 ± 4.06	**26.1 ± 12.6**
*Δcup*	2.43 ± 2.85	***8.61 ± 6.25***	1.24 ± 0.62	**4.91 ± 2.79**
*Δcup Δgig*	4.53 ± 2.20	4.66 ± 4.53	4.35 ± 1.41	10.6 ± 1.9
*Δcup Δcus*	4.35 ± 3.16	2.58 ± 2.65	4.19 ± 2.23	20.0 ± 13.5
*Δcop Δcup*	3.96 ± 2.04	***10.5 ± 4.9***	9.47 ± 5.69	9.37 ± 3.62
*Δcup Δcus Δgig*	5.12 ± 2.44	3.78 ± 2.42	3.95 ± 3.57	2.76 ± 2.84
*Δcop Δcup Δcus*	4.47 ± 1.42	6.18 ± 8.05	4.12 ± 2.43	***7.34 ± 3.73***
*Δcop Δcup Δcus Δgig*	3.86 ± 3.22	***9.04 ± 9.46***	5.92 ± 2.86	***10.0 ± 2.00***
*Δcop Δcup Δgig*	2.55 ± 0.99	4.85 ± 6.21	1.38 ± 0.64	**7.82 ± 3.97**

a The indicated mutant strains of *C. metallidurans* AE104 and the Δ*gshA* mutant were incubated for 20 h in the presence of Cu(II) provided at the IC_50_ concentration (Table 1) of the respective strain, without copper (negative control) and a Live/Dead staining was applied. The percentage of the dead cells (filter 3, λ_Ex_ 546 nm/λ_Em_ 590 nm) of the total cells (filter 2, λ_Ex_ 450 to 490 nm/λ_Em_ 520 nm) is given. Bold-faced values are Q > 1.5 and D > 1, *n* = 4 without copper and *n* = 6 with copper. Bold-faced and italic values have higher min, median and max values of these 4 or 6 experiments in the presence of copper compared to without copper but the mean values could not be judged as different due to a large deviation. Underlined values indicate a >50% increase in the presence of copper at the IC_50_ concentration but not judged as different due to large deviations. Light gray fields indicates no increased copper-mediated killing in the presence of GSH. Midgray field indicates neither in the presence nor the absence of GSH.

**TABLE 3:** Copper content of *C. metallidurans* mutant cells^*a*^

Bacterial strain	1,000 Cu/cell		Bacterial strain	1,000 Cu/cell	
Added Cu(II) (μM)	0	25	Added Cu(II) (μM)	0	25
*Δcus*	7.59 ± 2.64	49.6 ± 12.9	*Δcus ΔgshA*	5.13 ± 0.15	36.3 ± 5.7
AE104	5.97 ± 1.00	32.8 ± 5.6	AE104 *ΔgshA*	6.11 ± 1.41	23.0 ± 2.5
*Δgig Δcus*	7.33 ± 2.02	28.1 ± 3.0	*Δgig Δcus ΔgshA*	6.58 ± 0.74	25.3 ± 1.3
*Δgig*	7.01 ± 1.21	26.2 ± 2.7	*Δgig ΔgshA*	5.02 ± 0.47	24.4 ± 3.5
*Δcop Δgig*	5.67 ± 1.05	36.3 ± 7.5	*Δcop Δgig ΔgshA*	6.69 ± 0.41	**253 ± 17**
*Δcop*	7.83 ± 3.20	**52.4 ± 8.4**	*Δcop ΔgshA*	4.62 ± 0.67	**125 ± 25**
Added Cu(II) (μM)	0	1	Added Cu(II) (μM)	0	1
AE104	6.88 ± 1.29	20.5 ± 3.9			
*Δcop Δcus*	7.06 ± 1.33	26.0 ± 3.2	*Δcop Δcus ΔgshA*	5.00 ± 0.53	**45.5 ± 20.3**
*Δcop Δcus Δgig*	6.71 ± 2.21	30.6 ± 8.4	*Δcop Δcus Δgig ΔgshA*	4.71 ± 0.62	33.3 ± 12.4
*Δcup*	9.00 ± 3.41	19.9 ± 6.1	*Δcup ΔgshA*	5.50 ± 0.94	19.1 ± 2.6
*Δcup Δgig*	6.59 ± 2.29	20.6 ± 7.0	*Δcup Δgig ΔgshA*	6.02 ± 0.75	17.5 ± 0.8
*Δcup Δcus*	5.22 ± 1.16	20.3 ± 3.5	*Δcup Δcus ΔgshA*	5.09 ± 0.39	19.6 ± 1.1
Added Cu(II) (μM)	0	0.02	Added Cu(II) (μM)	0	0.02
AE104	6.92 ± 2.10	10.8 ± 0.8			
*Δcop Δcup*	6.48 ± 2.23	8.0 ± 1.0	*Δcop Δcup ΔgshA*	6.03 ± 0.58	13.6 ± 4.0
*Δcup Δcus Δgig*	6.11 ± 2.12	9.7 ± 0.3	*Δcup Δcus Δgig ΔgshA*	**3.30 ± 1.03**	8.0 ± 1.3
*Δcop Δcup Δcus*	8.04 ± 3.82	8.3 ± 3.2	*Δcop Δcup Δcus ΔgshA*	5.64 ± 0.44	9.8 ± 0.8
*Δcop Δcup Δcus Δgig*	6.32 ± 1.88	9.2 ± 0.3	*Δcop Δcup Δcus Δgig ΔgshA*	5.78 ± 1.10	12.0 ± 1.4
*Δcop Δcup Δgig*	5.85 ± 1.03	7.4 ± 2.6	*Δcop Δcup Δgig ΔgshA*	**3.68 ± 0.67**	11.5 ± 1.2

a The cells were cultivated in medium without (negative control) or with copper at the indicated concentrations. The copper content of the Tris-buffered mineral salts medium without added copper was 38 ± 23 nM (*n* = 4), as determined by ICP-MS. Cells were mineralized in 67% nitric acid at 70°C for 2 h. Samples were diluted to a final concentration of 2% nitric acid. Bold indicates ([Q ≥ 1.5 OR Q ≤ 0.66] AND D > 1) in the comparison to the parent strain AE104 cultivated in the presence of the same copper concentration.

**TABLE 4:** Glutathione content of *C. metallidurans* mutant cells^*a*^

Bacterial strain	nmol GSH/mg proteins	
Added Cu(II) (μM)	0	25
*Δcus*	**120 ± 38^*d*^**	**136 ± 42^*d*^^,^^*e*^**
AE104	593 ± 18	535 ± 73
AE104 *ΔgshA*	**−14 ± 7^*d*^**	ND
AE104 Δ*gshA*^*b*^	**−7 ± 3^*d*^**	ND
*Δgig Δcus*	536 ± 13	533 ± 73
*Δgig*	603 ± 2	540 ± 63
*Δcop Δgig*	593 ± 158	418 ± 88
*Δcop*	668 ± 45	553 ± 45
Added Cu(II) (μM)	0	1
AE104	593 ± 18^*c*^	505 ± 78
*Δcop Δcus*	608 ± 119	650 ± 81
*Δcop Δcus Δgig*	501 ± 38	**366 ± 19^*d*^**
*Δcup*	587 ± 14	576 ± 73
*Δcup Δgig*	422 ± 112	**662 ± 110^*f*^**
*Δcup Δcus*	487 ± 79	585 ± 77
Added Cu(II) (μM)	0	0.02
AE104	593 ± 18^*c*^	671 ± 83
*Δcop Δcup*	654 ± 182	552 ± 42
*Δcup Δcus Δgig*	498 ± 45	460 ± 45
*Δcop Δcup Δcus*	448 ± 80	548 ± 60
*Δcop Δcup Δcus Δgig*	403 ± 71	544 ± 82
*Δcop Δcup Δgig*	557 ± 98	630 ± 108

a Cells were cultivated in medium without (negative control) or with copper at the indicated concentrations and 5 mg cells were harvested, washed and disrupted by thaw-freeze cycling in liquid nitrogen. Protein content of the supernatant was determined with BSA as reference protein for a standard curve and GSH-content was determined by using the glutathione assay kit (CS0260, Sigma-Aldrich, Taufkirchen, Germany) with glutathione solution for the standard curve. ND; not determined.

b Deletion of *gshA* without markers.

c Same negative-control value for parent AE104 in all three parts of the table. Bold-faced numbers indicate differences ([Q ≥ 1.5 OR Q ≤ 0.66] AND D > 1) in comparison to: (i) AE104 without added copper; (ii) AE104 with the same concentration of added copper; or (iii) to the same strain without added copper.

d AE104 without added copper.

e AE104 with the same concentration of added copper.

f The same strain without added copper.

Copper resistance of these mutant strains was determined in dose-response experiments (Fig. S2) and used to calculate the IC_50_ values (Table 1). Copper resistance of these mutant strains was also compared with each other in other combinations to evaluate the effect of each system on copper resistance in *C. metallidurans* mutant strains (see Table S1 in the supplemental material). The Q value was the quotient of two IC_50_ values and the D value was the distance of their mean values divided by the sum of both deviations, as published (7, 33, 34). At *n* ≥ 3, D > 1 means a > 95% significance in the Student's *t* test. The residual resistance level of many multiple deletion mutants was about IC_50_ = 0.4 μM and this resistance level was referred to a “IC_50-min_.” The IC_50-min_ resistance level was reached by all mutants with Δ*cop Δcup* (Δ*cus* and/or Δ*gig*) mutations independent from the presence of glutathione, and additionally in Δ*gshA* mutants with the genotype Δ*cup* (Δ*cop* or Δ*cus Δgig*) (Table 1). Independent from the presence of glutathione, Cus or Gig were not able to mediate copper resistance above the IC_50-min_. In the absence of glutathione, Cop or Cus together with Gig also did not promote copper resistance. All five systems contributed to copper resistance and their functions were studied by the specific effect of each deletion in the parent and all mutant strains.

After the contribution of these systems to copper resistance has been outlined, the influence of the mutations on survival of the mutants and their metal and glutathione content was analyzed. Finally, the regulation of *cus* and *gig* was studied using a reporter gene system.

### Cup.

Deletion of *cup*, with the P_IB1_-type Cu(I)-exporting ATPase CupA as a central component, decreased copper resistance of the parent strain 46-fold. Cop, Cus, Gig, and GSH together were not able to fully substitute a missing Cup system. Copper resistance of the Δ*cup* single mutant was even lower than that of the Δ*cus Δgig*, Δ*cop Δgig*, and *Δcop Δcus* double mutants or the Δ*cop Δcus Δgig* triple mutant (Table 1), in the presence as well as in the absence of GSH. The impact of the combined Cop, Cus, and Gig systems and of GSH was smaller than that of Cup alone. This indicated the outstanding importance of Cup and sorted the mutants with respect to their copper resistance into two groups: (i) in the presence of GSH, the Cup-containing strains exhibited a mid to high degree of copper resistance with an IC_50_ > 150 μM; and (ii) all combinations of Δ*cup* mutants possessed only an IC_50_ < 15 μM (Table 1), a 10-fold difference in resistance. In the absence of GSH, all Δ*cup* mutants also had an IC_50_ value below 15 μM but the IC_50_ values of all Cup-containing strains was just above 30 μM (Table 1), a 2-fold difference. In the single comparison of all mutants with and without *cup*, the smallest decrease in the IC_50_ value was 34-fold (Δ*gshA ± *Δ*cup*; Table S1), while the largest of any non-Δ*cup* deletion in all the other mutants was 31.5-fold (Δ*cup ΔgshA* ± *Δcop*). Cup was by far the most important copper resistance system in *C. metallidurans*.

The strongest, 1,145-fold decrease in copper resistance resulted from deletion of *cup* in the Δ*cop Δgig* double mutant, which still contained Cus and GSH (Table S1). It went down to the IC_50-min_ value. The effect of a Δ*cup* deletion in a Δ*cop Δcus Δgig* background (which still contained GSH) was 336-fold and in a Δ*cop Δgig ΔgshA* background (which still contained Cus) 240-fold, leading to the IC_50-min_ value in both Δ*cup*-containing quadruple mutant strains. Cus was not able to substitute Cup in any way, not in the presence nor the absence of GSH.

The comparison of the effect of an additional Δ*cup* deletion in parent and single mutant strains yielded a ranking by the declining Q values of Δ*cus* (175-fold) > Δ*cop* (114-fold) > Δ*gig* (53-fold) > AE104 parent (46-fold) > Δ*gshA* (34-fold). Cup was more important in the absence of Cus or Cop than in that of Gig or GSH. Ranking of the declining Q values resulting from a Δ*cup* mutation in double mutants with a Δ*cop* or a Δ*cus* mutation led for the Δ*cop*-containing double mutants to Δ*cop Δgig* (1,145-fold to IC_50-min_) > Δ*cop ΔgshA* (383-fold to IC_50-min_) > Δ*cop Δcus* (243-fold to IC_50_ = 0.8 μM) > Δ*cop* single mutant (114-fold) and for the mutants with a Δ*cus* mutation Δ*cus Δgig* (269-fold) > Δ*cop Δcus* (243-fold) > *Δcus* single mutant (175-fold) > Δ*cus ΔgshA* (160-fold). Cup was more important in Δ*cop* mutants than in Δ*cus* mutants. The IC_50_ of 0.8 μM the Δ*cop Δcup Δcus* triple mutant, with a 2-fold IC_50-min_, was not different from the copper resistance of the quadruple Δ*cop Δcup Δcus Δgig* mutant (Fig. S2C, open diamonds compared to open inverted triangles) and the D value was just 1.03 (Table S1). Taking this into consideration, all Δ*cop*-containing double mutants went down to the IC_50-min_ by an additional Δ*cup* mutation. Cooperation of Cup and Cop was the most important contributor to copper resistance in *C. metallidurans*. Due to the increasing importance of Cup in double mutants with a Δ*cop* mutation, Cus, Gig, and GSH supported this Cup-Cop cooperation in the ranking of importance Gig > GSH > Cus.

Cup was the only system characterized here that was able to increase copper resistance of the quintuple mutant from the IC_50-min_ value 66-fold to IC_50_ = 29 μM (Δ*cop Δcus Δgig ΔgshA* mutant, Table 1). The task of the Cup system was unique, had a stronger impact on copper resistance than all the other systems combined, and could not be substituted by Cus or GSH, but needed cooperative interaction with Cop.

Since the Cup system is centered around the Cu(I)-exporting P_IB1_-type ATPase CupA, the unique and strong contribution of Cup to copper resistance would be the export of surplus cytoplasmic Cu(I) ions to the periplasm. Nevertheless, the other three systems and glutathione were also required to increase copper resistance in the presence of Cup from IC_50_ = 29 μM 21-fold to the resistance level of the parent strain, IC_50_ = 615 μM. If full copper resistance in *C. metallidurans* was considered the increase in the IC_50_ from the IC_50-min_ of the quintuple mutant, first Cup was needed (IC_50_ = 29 μM, *Δcop Δcus Δgig ΔgshA*), closely interacting with Cop (472 μM, *Δcus Δgig ΔgshA*), followed by contribution of Cus, Gig, and GSH (615 μM, parent). On the other hand, all strains with an IC_50-min_ resistance level were Δ*cup* mutants so that a Δ*cup* mutation was the first condition for a complete loss of copper resistance. Removal of surplus Cu(I) ions from the cytoplasm was an essential contribution to copper resistance, but alone, on the other hand, was not sufficient to reach full copper resistance in *C. metallidurans.* This phenotype resulted from an interplay of many systems.

### Cop.

Removal of the Cop system, centered around the periplasmic Cu(I) oxidase CopA (Fig. 1), resulted in a moderate 1.58-fold decrease in copper resistance (D = 2.04) in the parent strain background (Table 1). This effect of the *cop* deletion in the parent strain AE104 was well documented in the dose-response curves (Fig. S2A, closed diamonds versus circles). The other resistance systems were not fully able to substitute a missing Cop system but to 63% (1/1.58). Deletion of *cop* mediated a clear decrease in copper resistance in most mutants with the exception of the quintuple mutant and a very small 1.18-fold decrease (D = 1.09) in the *Δgig* single mutant (Table S1). Deletion of *cop* in all strains with a Δ*cup* deletion except the Δ*cup* single mutant resulted in the IC_50-min_, in agreement with a close cooperation of Cop and Cup. The resistance level of the Δ*cop Δcup* mutant was IC_50_ = 3.4 μM, 8.5-fold of the IC_50-min_, so that Cus, Gig, or GSH together could mediate some copper resistance. This was also demonstrated by the 925-fold decrease in copper resistance down to the IC_50-min_ that resulted from the Δ*cup* deletion in the *Δcus Δgig ΔgshA* mutant background, which still contained Cop.

Deletion of *cop* had the strongest effect (31.5-fold decrease) in the Δ*cup ΔgshA* background and led to the IC_50-min_ level (Table S1). This was the strongest effect of all non-Δ*cup* deletions. The decrease in resistance by deletion of *cop* was only 3-fold in the Δ*gshA* and only 4-fold in the Δ*cup* single mutants, indicating an important contribution of GSH to copper resistance to the function of the respective remaining system in the absence of Cup or Cop. A 26-fold decrease in the IC_50_-level resulting from the Δ*cop* deletion occurred in the Δ*cup Δgig* mutant, whereas a *cop* deletion in the Δ*gig* background had nearly no effect. Cup, Gig, and GSH were the most important interaction partners of Cop, but Gig only in the absence of Cup.

Cop was not able to increase copper resistance of the quintuple mutant but of all quadruple mutants. Cop in combination either with GSH, Gig, Cus, or Cup mediated a 5-fold, 9-fold, 13-fold or 16-fold increase in copper resistance, respectively. All four compounds led to an essential support to the Cop-mediated contribution to copper resistance in the ranking Cup > Cus > Gig > GSH. Cus, Gig, and GSH together, but not alone and not in single pairs, were able to mediate an IC_50_ = 3.4 μM. These three systems contributed to a function that could not be provided by any of the other two systems and only the resulting triple partner interaction network led to the emergence of a low degree of copper resistance. Together with Cup, these three systems were nearly able to substitute a missing Cop (63% of the IC_50_ of the parent strain, Table S1). Here, Cus and GSH were important, but not Gig.

Cop was second most important for copper resistance in *C. metallidurans*, but in contrast to Cup, Cop alone had no resistance effect; Cop needed the interaction with Cup, Cus, Gig, or GSH to mediate an increase in copper resistance in *C. metallidurans* above the IC_50-min_. Cooperation of Cup and Cop mediated 77% of the copper resistance level of the parent (*Δcus Δgig ΔgshA* compared with parent, D = 1.22) so that the combined contribution of Cus, Gig, and GSH to copper resistance was small (23%), but these systems together partially substituted a missing Cop. All Δ*cop Δcup* mutants with any other deletion (Δ*cus*, *Δgig* or *ΔgshA*) displayed a complete loss of copper resistance, so that Δ*cop* was the second most important condition for complete loss of this phenotype. On the other hand, a Δ*cup Δcus Δgig ΔgshA* quadruple mutant also had an IC_50_ = IC_50-min_, indicating again that Cop alone was not able to mediate any increase in copper resistance.

### Cus.

Deletion of *cus* in the parent strain had no effect. There was even a slight increase in copper resistance, which was well documented in the dose-response curves (see Fig. S2A in the supplemental material). This increase in resistance as consequence of Δ*cus* did not occur in the Δ*gig*, *Δcop*, or Δ*cup* single but in the Δ*gshA* double mutant (Table S1). A Δ*gig* deletion in the parent or the Δ*gshA* mutant abolished the Δ*cus*-mediated increase in copper resistance. Even in the presence of Cop and Cup, Gig supported copper resistance to a small degree.

Since deletion of *cus* in the parent strain had no effect, Cup, Cop, Gig, and GSH fully substituted a missing Cus system. Deletion of *cus* led to a 2-fold decrease of copper resistance in the Δ*cop* mutant (Fig. S2A), a 3-fold decrease in the Δ*cup* mutant, and a 4-fold decrease in the Δ*cup Δcop* mutant, down to the IC_50-min_ value (Table 1). Additional deletion of *gig* in the Δ*cop* and Δ*cup* single mutants led to a stronger decrease of copper resistance mediated by Δ*cus* as additional deletion in the double compared to the single mutants (Δ*cop ± *Δcus Q = 2 but Δ*cop Δgig ± *Δ*cus* Q = 3; Δ*cup ± Δcus* Q = 3 but Δ*cup Δgig* ± *Δcus* Q = 5; Table S1). Similarly, additional deletion of *gshA* led to a similar effect (Δ*cop ±* Δcus Q = 2 but Δ*cop ΔgshA ±* Δ*cus* Q = 6; Δ*cup ± Δcus* Q = 3 but Δ*cup ΔgshA* ± *Δcus* Q = 3.5; Table S1). Gig and GSH supported the contribution of Cus to copper resistance.

Deletion of *cus* decreased copper resistance in half of the mutant backgrounds (Table S1), in all mutants that carried a Δ*cop*, *Δcup*, or Δ*cup Δcop* mutation. Cus was partially able to support Cup in the absence of Cop as well as Cop in the absence of Cup, providing a back-up function for both systems. Cus was not able to raise copper resistance of the quintuple mutant or the quadruple mutants still containing Gig or GSH, but in the quadruple mutants possessing Cop or Cup. On the one hand, Cop and Cup fully substituted a missing Cus, on the other hand, Cus could also cooperate with either Cop or Cup to mount a moderate degree of copper resistance. The strongest decrease in copper resistance as result of a Δ*cus* deletion occurred in the Δ*cup Δgig ΔgshA* mutant, 12-fold down to the IC_50-min_, with a smaller effect in the Δ*cup Δgig* (5-fold) and the Δ*cup ΔgshA* (6-fold) but none in the Δ*gig ΔgshA* background.

Cooperation of Cup, centered around the P_IB1_-type ATPase CupA, and of Cop, centered around the periplasmic Cu(I) oxidase CopA, was central to copper resistance in *C. metallidurans*. Cus, centered around the transenvelope efflux complex CusCBA, was not required when Cup and Cop were present but was required when either system was absent. The absence of copper resistance above the IC_50-min_ value in the quadruple mutant with Cus as sole component clearly demonstrated that CusCBA was not able to substitute the function of CupA as efflux pump for cytoplasmic Cu(I) ions. Instead, substitution of CopA gave evidence that CusCBA removed periplasmic Cu(I) ions by export to the outside while CopA oxidized these to Cu(II) ions. Either way resulted in decreased uptake of Cu(I) into the cytoplasm and consequently relief the necessity of CupA (Fig. 1).

### Gig.

Deletion of *gig* had no effect in the parent, so the other systems were fully able to substitute a missing Gig system. The effect of a *gig* deletion in all strains with a functional Cup system was small (1 > Q > 2, D > 1, mutants Δ*cus* ± Δ*gshA*, Δ*cop* Δ*gshA*, Δ*cop Δcus*) or not existing (D < 1, Δ*gshA*, Δ*cop Δcus* Δ*gshA*, Table S1), indicating a minor contribution of Gig in Cup^+^ strain with additional possession of Cop, Cus without GSH, and GSH without Cus but not with Cus and GSH. There was no effect of the *Δgig* deletion in the Δ*cup* mutant but in the *Δcup* mutants with an additional Δ*gshA* ± Δ*cus* or Δ*cop* deletion. The strongest decrease in resistance (7-fold to 8-fold down to the IC_50-min_) occurred in the Δ*cop Δcup* double and in the Δ*cup Δcus ΔgshA* triple mutant. Contribution of Gig to copper resistance was visible in a Δ*cup* background with additional absence of Cop or of Cus plus GSH.

Gig was not able to increase copper resistance of the quintuple mutant, not in quadruple mutants still containing Cus, GSH, or Cup but in the quadruple mutant still containing Cop. Gig cooperated with Cop to increase copper resistance of the quintuple mutant to a moderate degree of IC_50_ = 3.8 μM, with Cus plus GSH to a similar level (IC_50_ = 3.4 μM), and doubled copper resistance in cells having either GSH and Cop, or Cus, and Cop.

The contribution of Gig to copper resistance was visible in cells with impaired removal of excess cytoplasmic (Cup) and of periplasmic (Cop, Cus) Cu(I) ions. In multiple mutants, Gig was required for full function of Cop and of Cus plus GSH.

### GSH.

Absence of GSH had a small (1 < Q < 2, D > 1) effect in the parent strain, the Δ*cus*, *Δgig*, or *Δcus Δgig* mutants (Table 1). Contribution of GSH to copper resistance was small in cells with Cup and Cop systems, which together largely substituted missing GSH. Copper resistance decreased as result of a Δ*gshA* mutation in Δ*cop* ± Δ*cus* ± Δ*gig* strains and in Δ*cup* mutants with an additional deletion of Δ*gig* (moderate effect), Δ*cop*, *Δcus Δgig*, or Δ*cop Δcus* (moderate effect) but not in the *Δcup* single mutant. GSH was not able to substitute a missing Cup system but was needed when Cop, Gig, or Cus were also absent in the Δ*cup* mutant. The strongest decrease in copper resistance mediated by Δ*gshA* occurred in the Δ*cop Δcus* (7-fold) and the Δ*cop Δcup* (8-fold) mutants. In Δ*cop Δcup*, any additional deletion yielded the IC_50-min_. As judged by the increase in the Q value following deletion of *gshA*, importance of GSH increased from Δ*cop* (2.4-fold) to Δ*cop Δgig* (4.5-fold), Δ*cop Δcus* (7.4-fold) with a decrease of 5.4-fold when *gshA* was deleted in the Δ*cop Δcus Δgig* mutant, indicating a contribution of GSH to Gig- and Cus-mediated substitution of Cop.

Presence of GSH was not able to increase copper resistance of the quintuple mutant, quadruple mutants still containing Cus, or Gig, but those with a remaining Cup or Cop. GSH alone was not able to mediate copper resistance but GSH increased Cup-mediated copper resistance 5-fold, was essential for a Cop-mediated 4-fold-, and a Cus-Gig-mediated 8-fold increase above the IC_50-min_. GSH could not increase copper resistance when Cop and Gig or Cop, Gig, and Cus were present, but to a small part (Q = 1.77, D = 1.52) when only Cop and Cus were present but Gig was absent.

The absence of GSH in the *Δcup* mutant, which lacked the major efflux system CupA for cytoplasmic Cu(I) ions but possessed Cop, Cus, and Gig, indicated that a Cu(I)-buffering activity of GSH in the cytoplasm did not contribute to copper resistance in *C. metallidurans*. Instead, GSH became important when Cop was missing in the parent or Δ*cup* background. GSH supported copper resistance based on the CopA-mediated oxidation of Cu(I) in the periplasm. Lack of Cus and Gig increased importance of GSH for this process. Moreover, GSH even allowed a 4-fold increase of copper resistance above the IC_50-min_ by an interplay of GSH with Cop, and an 8-fold increase by an interplay with Cus and Gig.

### Interaction of the five systems.

Cup, centered around the P_IB1_-type ATPase CupA, which exports excess Cu(I) from the cytoplasm, was by far the most important copper resistance system in *C. metallidurans*. Nevertheless, full copper resistance required cooperation of Cup with Cop. Cus, Gig, and GSH, forming a triple-partner interaction network, supported the Cup-Cop cooperation. The Cop system, centered around the periplasmic Cu(I)-oxidase CopA, needed the interaction with Cup and with Cus-Gig-GSH. Any decline of the oxygen tension may impair Cop and enhance the importance of Cus, Gig, and GSH as substitute for the interaction with Cup. Cop and Cup fully substituted a missing Cus, but Cus could also cooperate with either Cop or Cup to mount a moderate degree of copper resistance. Cus alone was not able to substitute Cup in any way, not in the presence nor the absence of GSH, so the transenvelope efflux complex CusCBA should not be able to export cytoplasmic Cu(I) ions. The contribution of Gig to copper resistance was visible in cells with impaired removal of excess cytoplasmic (Δ*cup*) as well as of periplasmic Cu(I) ions by CopA or CusCBA. Gig supported the function of Cop and of Cus plus GSH. A potential Cu(I)-buffering activity of GSH in the cytoplasm did not contribute to copper resistance. GSH increased copper resistance by an interplay of Cup, Cop, with Cus plus Gig, or to a moderate degree when Cop and Cus were present, but Gig was absent. Full copper resistance in *C. metallidurans* results from an interplay of these five systems.

### Copper kills.

Live/Dead staining followed by microscopy was used to investigate whether the copper has a more bactericidal or bacteriostatic effect on multiple deletion strains (Table 2, Fig. S3 and S4). The cells were incubated in the presence and absence of Cu(II) in the growth medium. The IC_50_ value of the individual strain was used as copper concentration (Table 1). This allowed comparison of strains with IC_50_ values of copper between 743 μM and the IC_50-min_ of about 0.4 μM. These concentrations should have the same physiological impact on the compared strains. A bactericidal effect should increase the percentage of dead cells in the culture compared to the control without copper, and the deviation of the results since the difference in turbidity of individual cultures in biological repeats of dose-response experiments was always larger close to the IC_50_ value than at lower or higher copper concentrations (Fig. S2). Intact cells displayed a green fluorescence when filter 2 was used while membrane-damaged cells fluoresced in red (filter 3; an example is shown in Fig. S3). From the images, the percentage of dead cells was calculated. Isopropanol-treated cells served as negative control and were all dead (Table S2). Not only the mean values of the percentage of dead cells plus its deviation was calculated but also the minimum, median and maximum value from the biological repeats (Table S3). These values served also to judge the impact of copper at the IC_50_-concentration on the cells (Table 2).

In GshA^+^ cells (Table 2), the mean value of the percentage of dead cells in the absence of copper ions was 3.77 ± 0.98%. About 4% of the cells in a *C. metallidurans* culture were always dead. Mutations in the copper resistance systems did not change this value in the absence of copper ions. When the cells were incubated with copper added at the concentration of their individual IC_50_ for copper, large differences between the individual cultures led to strong deviations of percentage of dead cells (Table S2), leading to low D values (Table 2), as expected. The increase of the percentage of dead cells was also counted as “different” compared to the control without copper when the minimum, median, and maximum values were all three higher in copper-treated cells compared to the control (Table S3, bold-faced and italic values in Table 2) or at least the mean value was 50% higher (underlined values in Table 2). In the parent strain and most mutants, treatment with copper at the IC_50_ concentration increased the percentage of dead cells so that copper ions indeed killed a larger part of the population than in control cells. This effect could also be observed with only the IC_50-min_ concentration of 0.4 μM Cu(II) and the Δ*cop Δcup Δcus Δgig* quadruple mutant, albeit with huge deviations of the measurements between the individual biological repeats. Even very low copper concentrations were able to kill bacteria that did not possess copper resistance systems.

In Δ*gshA* cells (Table 2), this pattern was not different. Between 1.2% and 6.5% of the cells were dead even in the absence of copper ions, and these values increased when copper was added. The highest percentage of killed cells (31%) were reached in the *Δcop Δgig ΔgshA* mutant, indicating that Cop, Gig, and GSH were important contributors with respect to protection of the cells against copper-mediated killing.

Several mutant strains were not killed by copper at their respective IC_50_ values. In the Δ*cup Δgig* and the Δ*cup Δcus* mutants, presence of copper did not increase the percentage of dead cells in GSH^+^ cells but did increase in the corresponding Δ*gshA* mutants (Table 2, light gray field). In the Δ*cup Δcus Δgig* triple mutant, presence of copper did not lead to increased death ratios, even in the absence of GSH (Table 2, medium-gray field). The respective Δ*cup Δcus Δgig ΔgshA* quadruple mutant contained the Cop system as the sole remaining system, and had an IC_50_ equal to the IC_50-min_. This copper concentration did not increase killing of this quadruple mutant but clearly increased killing of the quintuple and several other mutants at the same copper concentration. Cop alone protected the cells against copper-mediated increased killing in the Δ*cup Δcus Δgig ΔgshA* mutant at the IC_50-min_. The additional presence of GSH (Δ*cup Δcus Δgig* mutant) allowed an IC_50_ of 2 μM and Cop was still able to protect the cells. GSH allowed the cells to survive and grow at a 4.35-fold higher concentration than the IC_50-min_ by protecting them against copper-mediated killing in cooperation with Cop, which defines a role of GSH in copper resistance.

When, in the next step, presence either of Cus or of Gig mediated an IC_50_ between 4 μM and 11 μM, protection by Cop required the presence of GSH. Cus and Gig were able to support Cop and GSH in their respective protecting action in the Δ*cup Δgig* or Δ*cup Δcus* double mutants. When Gig was added to the Δ*cup Δgig* mutant or Cus to the Δ*cup Δcus* mutant, this resulted in the Δ*cup* single mutant with an IC_50_ of 13.5 μM, which was killed by addition of this copper concentration, although there was no difference between the copper resistance levels of the Δ*cup* single and the Δ*cup Δgig* double mutant. When Cup was added to the Δ*cup Δgig* or the Δ*cup Δcus* mutants, parent resistance levels were reached, and the cells were inactivated by copper at the respective IC_50_ value.

The Cop system, centered around the periplasmic Cu(I) oxidase CopA, contributed to copper resistance in *C. metallidurans* by protecting the cells against copper-mediated killing at the IC_50-min_, although the Cop system did not increase copper resistance above the IC_50-min_ in the respective Δ*cup Δcus Δgig ΔgshA* quadruple mutant. GSH alone did not protect the cells against copper-mediated killing at the IC_50-min_ in the Δ*cop Δcup Δcus Δgig* quadruple mutant but Cop plus GSH protected and increased the IC_50_ level 4-fold in the Δ*cup Δcus Δgig* triple mutant. Further addition of Gig (Δ*cup Δcus* double mutant) or Cus (Δ*cup Δgig* double mutant) increased the resistance level of the protected cells a second time (IC_50_ = 4 μM and 11 μM, respectively) but only in the presence of GSH. Cop-GSH mediated a first layer of protection against copper-mediated killing and Gig or Cus built upon this. Addition of Cus and Gig to the Δ*cup Δcus Δgig* triple mutant increased the IC_50_ 5-fold to 13 μM but the cells were not protected any more against killing at this copper concentration so that the effects of Gig and Cus were additive with some overlap with respect to resistance but counteracting with respect to Cop-GSH-mediated protection against killing. Similarly, any addition of Cup strongly increased the copper concentration that could be tolerated, which nevertheless increased the number of cells that were killed by copper.

### Copper content of the mutant cells.

In general, the cellular copper and metal content of the mutant cells was determined in the presence and absence of copper ions added to the growth medium. Since dead cells hyperaccumulate copper ions (35) and most copper-treated mutant cell populations contained an increased number of mutant cells (Table 2), the copper concentrations used in these experiments were well below the IC_50_ values (Table 1). For cells with and without *gshA* with an IC_50_ > 100 μM, a concentration of 25 μM added Cu(II) was used, for cells with and without *gshA* with an 100 μM > IC_50_ > 1 μM, a concentration of 1 μM added Cu(II) was used, and for the remaining cells, a concentration of 0.02 μM (Table 3). This careful approach not only eliminated artifacts resulting from unspecific binding of copper to dead cells but also led to a high resolution of the effects of the single copper resistance systems on copper resistance and accumulation at low copper concentrations, e.g., those that allowed Cop-GSH to protect the cells against copper-mediated killing.

All cells cultivated without added copper contained about 6,000 atoms of Cu per cell (Table 3), caused by the 38 ± 23 nM Cu(II) in the growth medium. Only two quadruple mutants cultivated in medium without added extra copper exhibited a lower cellular copper concentration of about 3,500 Cu per cell. Since one of these strains contained 4,710 ± 620 Cu per cell when it served as negative control for cells cultivated in the presence of 1 μM added copper instead of 20 nM added copper (Table S4), both low values were irrelevant fluctuations and not further considered.

The AE104 parent cells increased their copper content in the presence of 25 μM copper to about 33,000 Cu atoms per cell, in the presence of 1 μM Cu to 20,000 Cu atoms per cell, and in the presence of 20 nM Cu to 10,000 Cu atoms per cell, showing a saturation curve of the amount of cell-bound copper instead of a linear relationship. None of the multiple mutant strains incubated with 20 nM added copper showed an increased cellular copper content compared to the parent cell, although all these mutants carried a Δ*cup* deletion. This included the *Δcup Δcus Δgig* and *Δcup Δcus Δgig ΔgshA* mutants, which were protected by Cop against copper-mediated killing at the IC_50-min_ and in the presence of GSH at an IC_50_ = 2 μM. At 1 μM added copper, none of the Δ*cup* strains displayed an increased cellular copper content. This included the Δ*cup* Δ*gig* and the Δ*cup Δcus* double mutants, which were protected by Cop-GSH against killing at their IC_50_ of 11 μM and 4 μM, respectively. This indicated that *cup* with the P_IB1_-type ATPase CupA as the central component was not required to adjust the cellular copper content at 1 μM added copper. Second, the protecting effect of Cop, GSH, Cus, or Gig was not accompanied by a decreased cellular copper content at low copper concentrations.

At 25 μM copper, the copper content of the Δ*gshA* single mutant was slightly below that of the parent cell (23,000 Cu per cell versus 33,000 Cu per cell, Q = 0.70, D = 1.21), so that presence of GSH mediated a small increase in the cellular copper content. The presence of GSH alone did not increase copper resistance of the quintuple mutant (Table 1). The copper content of the Δ*cup Δgig* and Δ*cup Δcus* mutants plus or minus GSH was not different (Table 3) although GSH was essential for the Cop-mediated protection against copper-caused enhanced killing (Table 2) at 2 μM added copper. Since GSH, as the main cellular thiol compound, is required for repair of oxidative damage, this all means that a GSH-mediated increase in copper resistance by interaction of GSH with the other resistance systems was not based on complexation of copper by GSH but rather by repair of copper-mediated oxidative damage.

While the cellular copper content of the parent cells at 25 μM added copper was 33,000 Cu per cell, the Δ*cop* mutant contained 52,400 Cu atoms per cell (Table 3). This demonstrated that Cop, which was no longer able to protect the cells at this concentration against copper-mediated killing, nevertheless contributed at these copper concentrations to copper resistance by causing decreased accumulation of copper, even in the presence of Cup. Consequently, Cop interacted with GSH and subsequently Gig and Cus to prevent copper-mediated killing at low concentrations, and with Cup to prevent accumulation of copper at high concentrations.

To add another layer to this interaction network, Gig, Cus, and GSH influenced the Cop-Cup-mediated decrease in cell-bound copper (Table 3). In the presence of Cus and at 25 μM added copper, the Δ*cop* Δ*gshA* cells contained the 2.5-fold amount of cell-bound copper compared to that of the *Δcop* single mutant. Additional deletion of *gig* doubled the number of cell-bound copper again, while this number was not increased compared to that of the parent strain AE104 in the Δ*cop Δgig* mutant. GSH quenched copper accumulation in Δ*cop* Cus^+^ Cup^+^ cells by allowing Cus or Cup to export copper ions. Gig was able to substitute GSH to some extent in these cells. Since the IC_50_ values of the Δ*cup*, the *Δcup ΔgshA*, and the Δ*cup Δgig* strains were not different (Table 1), Cup did not need GSH or Gig for function so that the underlying interaction network is the Cus-Gig-GSH triangle, which also caused a small increase in copper resistance compared to the quintuple mutant. In the presence of GSH, Gig seemed to stimulate copper accumulation in Δ*cop* cells but this effect was close to the significance threshold when the copper contents of Δ*cop Δgig* and Δ*cop* cells were directly compared with each other (Table 3, Q = 1.44, D = 1.01), whereas the comparison of the copper contents of the Δ*cop ΔgshA* ± *gig* cells yielded a significant result (Table 3, Q = 2.02, D = 3.05). GSH quenched copper accumulation in Δ*cop* cells by interacting with Cus. Gig could substitute GSH here to some part.

At 1 μM added copper, the copper content of the Δ*cop Δcus Δgig* and Δ*cop Δcus* cells was not different from the parent (Table 3). There was some increase in the Δ*cop Δcus ΔgshA* mutant compared to the parent but this comparison possessed only a D value close to the significance threshold due to a large deviation of one value (Q = 2.22, D = 1.03). No difference could be observed between the copper contents of the Δ*cop Δcus* ± Δ*gshA* cells (Q = 1.75, D < 1) and of the Δ*cop Δcus ΔgshA* ± *Δgig* cells (Q = 1.37, D < 1). At 1 μM added copper, neither Cup nor Cop were required to limit accumulation of copper by the cells and consequently, Cus-Gig-GSH were not needed to substitute a missing Cop.

### Glutathione.

Marker-free deletion or interruption of the *gshA* gene in *C. metallidurans* resulted in a decrease of the measured cytoplasmic GSH in wild-type cells from 600 nmol GSH per mg protein to GSH values that could no longer be detected (Table 4, negative values). This cellular GSH concentration corresponds to 42 million GSH molecules per cell (Table 4), so that the slightly increased copper content of 10,000 atoms per cell represents only a neglectable portion of the total GSH pool that was occupied by copper ions.

The GSH content of the cells with the deletions of copper resistance determinants was not different from that of the parent strain AE104 in cells incubated without added copper (Table 4) with exception of the Δ*cus* mutant. This strain exhibited only 20% of the GSH content of the parent. Any additional deletion (Δ*gig Δcus*, *Δcop Δcus*, *Δcup Δcus*) increased the GSH content back to the parent level. The decreased GSH content in the Δ*cus* strain depends on the presence of Gig, Cop, or Cup. In the presence of copper, added again at the same concentration used for the determination of the cellular copper content, the GSH level of the *Δcus* strain was also decreased.

The GSH content of mutant strains incubated in the presence of 1 μM or of 20 nM added copper was not different from that of the parent or the same mutant cultivated without copper (Table 4), with two exceptions. The GSH content of copper-treated cells was decreased in the *Δcop Δcus Δgig* triple and increased in the *Δcup Δgig* double mutant but not in the respective Gig^+^ strains, which were also incubated with 1 μM copper. Gig prevented a decrease in the GSH level in the Δ*cop Δcus* but an increase in the Δ*cup* strain. Gig seems to influence the GSH level in *C. metallidurans*.

### Regulation of the *cus* determinant.

**FIG 2:**
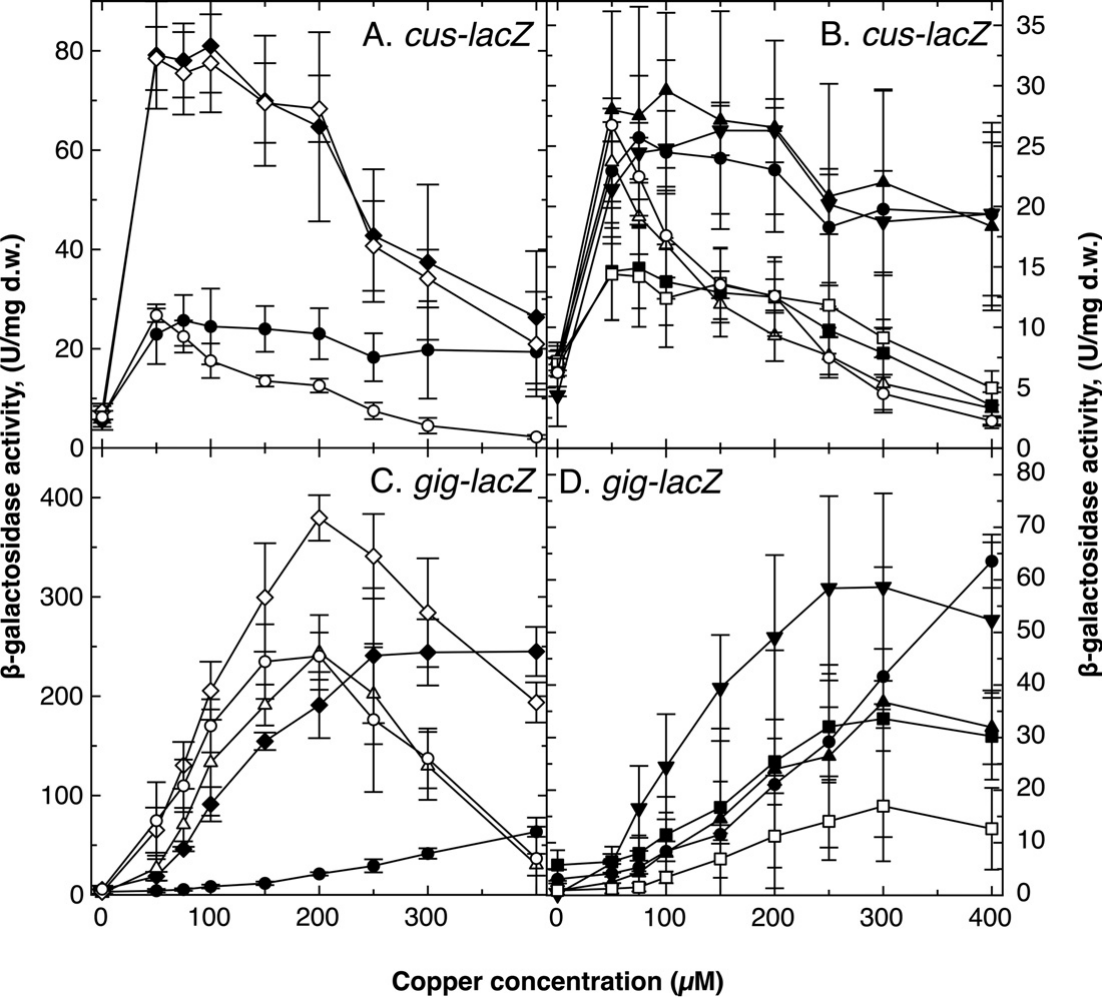
Regulation of *cus* and *gig*. Reporter gene fusions with the *lacZ* gene were constructed with the *cus* (A and B) and *gig* operons (C and D) in various mutant backgrounds. The strains were incubated in the presence of increasing copper concentrations and the beta-galactosidase activity was determined. A to D: strain AE104 (closed circles, ●), Δ*cop* (closed diamonds, ◆), Δ*gshA* (closed inverted triangles, ▾), Δ*cop Δcup* (open triangles, △), Δ*cup* (closed squared, ◼). A and B: Δ*gig* (closed triangles, ▴), Δ*cop Δgig* (open diamonds, ◇), Δ*cop Δcup Δgig* (open circles, ○), Δ*cup Δgig* (open squares, ☐). C and D: Δ*cus* (closed triangles, ▴), Δ*cop Δcus* (open diamonds, ◇), Δ*cop Δcup Δcus* (open circles, ○), Δ*cup Δcus* (open squares, ☐). Deviations shown (*n* ≥ 3).

A *lacZ* reporter gene was inserted downstream of the *cusDCBAF* genes on the chromid of a variety of *C. metallidurans* AE104 mutants. The cells were incubated in the presence of increasing copper concentrations and the reporter activity was measured (Fig. 2A and B). In the parent strain AE104, *cus-lacZ* was upregulated by 50 μM copper to a specific activity of about 23 ± 8 U/mg dry mass and remained on this level with increasing copper concentrations. In the Δ*cop* and Δ*cop Δgig* mutants, the activity reached the 4-fold specific activity at 50 μM copper and decreased with increasing copper concentrations to the level of the AE104 parent cells of 20 U/mg at 400 μM Cu(II). This copper concentration was in the same range as the IC_50_ values of both strains.

In total, four different modes of copper-dependent *cus*-activation were evident: first, the strong activation by copper in the Δ*cop* ± *Δgig* mutants (Fig. 2A); second, activation similar to the parent cells in Δ*gig* and Δ*gshA* cells; third, activation similar to the parent up to 50 μM added Cu(II) but a rapid decline at higher concentration in Δ*cop Δcup* ± *Δgig* cells; and last, a lower level of activation at 50 μM Cu(II) followed by declining expression levels in Δ*cup* ± Δ*gig* cells. Since the IC_50_ values of all Δ*cup* cells were ≤ 13.5 μM, this decline was probably the result of copper-mediated inhibition of the cells. Gig and GSH were not involved in regulation of *cus*. The strong activation in all *Δcop* mutants with lacking periplasmic Cu(I) oxidase CopA indicated that periplasmic Cu(I) ions were most likely the regulator of *cus* expression.

### Regulation of the *gig* determinant.

**FIG 3:**
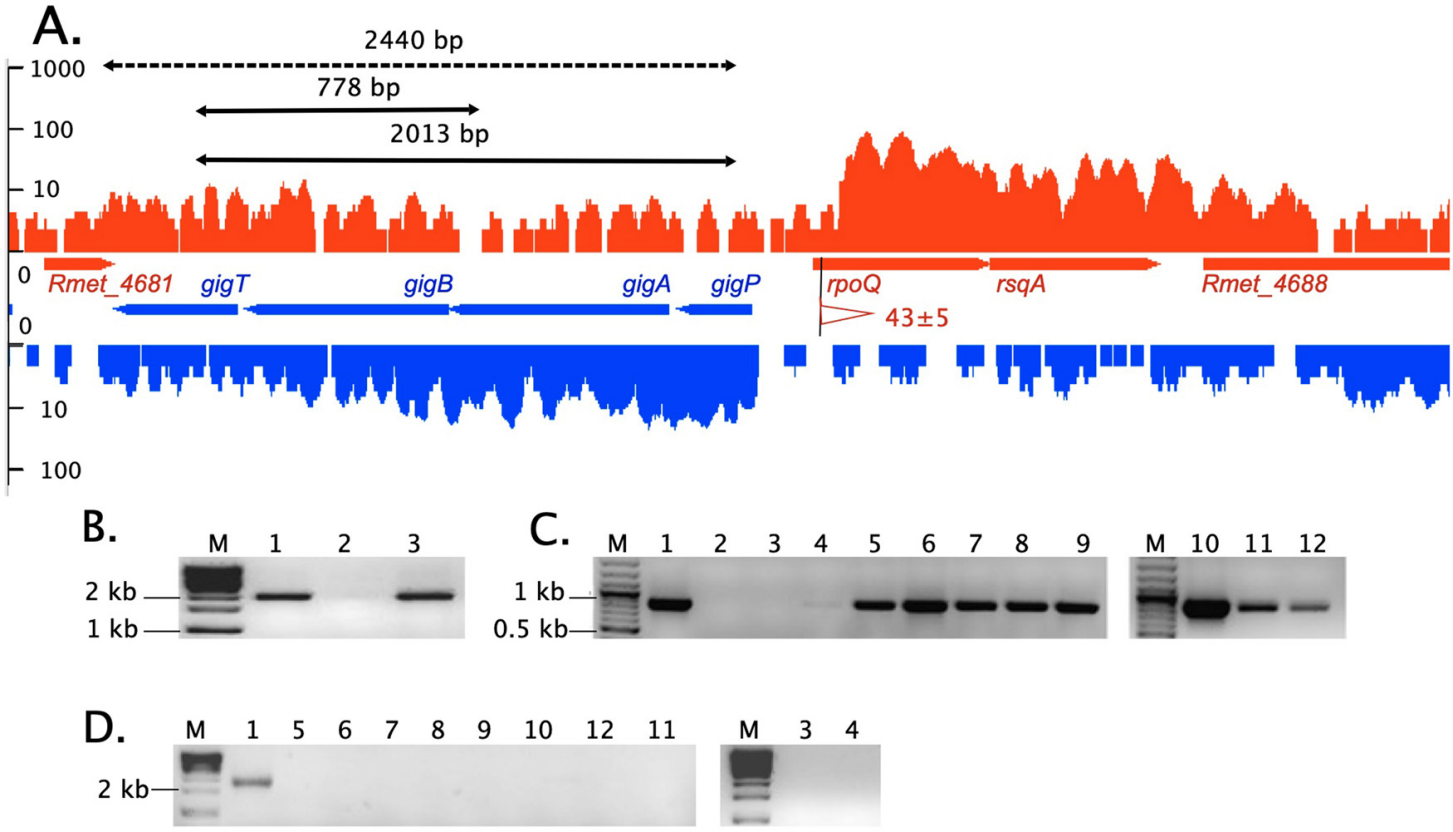
The *gigPABT*-*rpoQ-rsqA* gene region on the chromid of *C. metallidurans.* (A) As part of Fig. S1A, the open reading frames for the genes *Rmet_4681*, *gigPABT*, *rpoQ*, *rsqA*, and *Rmet_4688* and the corresponding transcriptional activity in *C. metallidurans* wild-type cells as nucleotide activities per kilobase of exon model per million mapped reads (NPKM) values are shown in red (plus strand) or blue (minus strand) on a logarithmic scale (100). This region corresponds to base pairs 1.313.350 to 1.317.350 on the chromid (accession number CP000353). A transcriptional start site with a low TSS score (<50) is shown as a flag with the red color indicating dependence on RpoD (103). No transcriptional start site was found upstream of *gigP* under nonchallenging conditions. The two-sided arrows on the top show the result of an RT-PCR experiment with positive (solid line) or negative (dashed line) outcome. The RT-PCR results are shown in panels B to D with the marker (M) always shown on the left hand. (B) (2,031-bp fragment, primers *gigT*>*gigP*): 1, positive-control DNA; 2, negative-control water; 3, RNA from cells incubated in the presence of 25 μM Au(III). (C) (827 bp, primers *gigT>gigA*). (D) (2,458 bp, primers Rmet_4681>*gigP*): 1; positive and 2 negative control (not shown in panel D). Lanes 4 to 12 RT-PCR with RNA from cells incubated in the presence of various conditions: 3, no metal; 4 to 8 Au(III) (4, 30 min 10 μM; 5, 10 min 25 μM; 6, 30 min 25 μM; 7, 10 min 50 μM; 8, 30 min 50 μM); 9 to 12 Cu(II) (9; 10 min 100 μM; 10, 30 min 100 μM; 11, 30 min 500 μM; 12, 10 min 500 μM). Please note that the lanes in panel D, which correspond to that in panel C, are in a different order. The PCR results clearly prove the existence of the predicted *gigPABT* operon Op1355r_1.

RT-PCR experiments verified the operon structure and regulation of *gig* transcription (Fig. 3). The *gigPABT* genes formed an operon from upstream of *gigP* to downstream of *gigT* but not beyond. These experiments also demonstrated that copper induced transcription of the *gig* (gold induced) genes as well as gold complexes.

A *lacZ* gene was inserted downstream of *gigT* and the activity of the *gig-lacZ* fusion was determined at increasing copper concentrations (Fig. 2C and D). In parent strain AE104, *gig-lacZ* activity increased with increasing copper concentrations to a specific activity of 67 U/mg. In a Δ*cop* strain (Fig. 2C, closed diamonds), activity reached 250 U/mg at 250 μM copper and remained there up to a copper concentration of 400 μM (Fig. 2C). There was no decline of the reporter activity in the Δ*cop* mutant with an IC_50_ of around 400 μM at this concentration, so the decline of the *cus-lacZ* fusion (Fig. 2A) was not the result of copper-mediated inhibition of translation but of a decreased upregulation of *cus*. The strong expression in Δ*cop* cells indicated that *gig* was also regulated by periplasmic Cu(I) ions. Moreover, Gig was needed at higher copper concentrations than Cus.

Expression of *gig-lacZ* in the Δ*cop Δcus* double mutant was even stronger than in the Δ*cop* mutant and peaked at 200 μM added Cu(II). Since *gig* was regulated by periplasmic Cu(I) ions and its expression increased following deletion of *cus* in Δ*cop* mutants, this was evidence that Cus indeed decreased the periplasmic Cu(I) level by CusCBA-mediated copper efflux to the outside.

Expression of *gig-lacZ* in Δ*cop Δcup ± Δcus* cells also increased at copper concentrations up to 200 μM added Cu(II) and decreased thereafter, probably due to the lower copper tolerance of the Δ*cup* deletion strains. Single mutants with a Δ*cup* or a *Δcus* deletion regulated *gig-lacZ* similar to the parent (Fig. 2D) but decreased expression at 400 μM Cu(II). In the presence of Cop, a *Δcus* deletion no longer resulted in an upregulation of *gig*. Absence of GSH in the *ΔgshA* strain led to an upregulation of *gig-lacZ*.

## DISCUSSION

### The cellular stage for copper homeostasis.

Homeostasis of copper as an “essential-but-toxic” element is an important cellular process in many organisms. Consequently, copper resistance systems are widespread in cultivated bacteria and in complex communities in many ecosystems. Here, most important are Cup- and Cus-like systems (36). Copper tolerance is a virulence factor (37). It is not only involved in the interaction of pathogenic bacteria with a human host but also for survival of bacteria within amoeba (38), indicating an ancient origin of the strategy to use copper ions as “war heads” against intruding bacteria. This all points out the necessity to understand the full picture of copper toxicity, homeostasis, and resistance. These processes involve multiple pathways (39) and our publication describes that copper resistance is an emergent feature based on the interaction of several resistance and tolerance mechanisms, some of them acting in the periplasm of Gram-negative bacteria.

For cells growing under oxic conditions, copper is usually present as Cu(II) ion. The redox potential of the Cu(II)/Cu(I) at pH 7 is E_o_’ = −267 mV (40). Cu(II) may pass the outer membrane of *C. metallidurans* and other Gram-negative bacteria by facilitated diffusion across unspecific porins or by TonB-dependent active transport across this membrane (1, 41–44). In the periplasm, Cu(II) can be reduced by contact with respiratory chain components (45) or by reducing equivalents provided by a cysteine-cystine shuttle (46), probably by electrons stemming from NADH (E_o_’ = −320 mV) (47). Several pathways allow import of copper ions from the periplasm into the cytoplasm. Cu(II) can be imported as an unspecific substrate, for instance by the Zn(II)-importer ZupT of the ZIT/IRT (ZIP) transporter family (48). Subsequently, incoming Cu(II) should be immediately reduced to Cu(I) by a rapid, thiol-mediated process (28).

Copper is more toxic to E. coli under anaerobic conditions compared to aerobic conditions (10). Export of cytoplasmic copper in E. coli depends on a CupA-orthologue. Expression of the respective gene is under the control of the MerR-type regulator CueR with its zeptomolar Cu(I) affinity (49). The time-dependent profiles of the CueR-dependent expression of its target-genes clearly indicate that much more Cu(I) arrives in the cytoplasm under anaerobic conditions than under aerobic conditions, leading to increased anoxic copper sensitivity (10). An even lower resistance level occurs in E. coli cells growing in the presence of substrates that are imported by Na(I)-dependent import systems. This indicates that a third, unspecific Cu(I) import system exists in bacteria that may use Na(I)-dependent importers such as MelB. Na(I) binds to copper-binding proteins and copper ions inhibit Na(I) transport across membranes (50). *C. metallidurans* can survive anoxic conditions by using nitrate respiration (51). Mutants of *C. metallidurans* carrying multiple deletions in the genes for metal uptake systems nevertheless import metal ions by an unknown high-rate importer, which also transports Cu(I) and even gold ions into the cytoplasm (8, 34, 52, 53). Cu(I) is a better substrate for import into the cytoplasm than Cu(II) and unspecific high-rate uptake systems, e.g., by Na(I)-dependent transporters, may be responsible for Cu(I) import into the cytoplasm.

In Gram-negative bacteria such as E. coli, DNA is not damaged by Cu(I)-catalyzed oxidation in the presence of GSH since most of the hydrogen peroxide-oxidizable copper is located in the periplasm. Most of the copper-mediated hydroxyl radical formation occurs in this compartment (54), which affects processes here such as the assembly of c-type cytochromes (55). In the cytoplasm, one of the Cu(I) targets are FeS-clusters. FeS-dependent dehydratases are inhibited (56) and FeS-cluster assembly is interrupted (57–59), e.g., by binding of Cu(I) to IscA (60). Due to the higher reduction rate of Cu(II) to Cu(I) and the resulting higher import rates of copper ions, this leads to strong copper-mediated inhibition under anoxic conditions. The lower iron content of cells with deleted copper resistance determinants plus a Δ*gshA* mutation, especially in the presence of copper ions, indicates that iron homeostasis of *C. metallidurans* is also affected by copper and that GSH protects to some part against this damage (Table S4). Moreover, and reminiscent to Cd(II), Cu(I) strongly binds to thiol residues, leading to damaged proteins (61, 62) and upregulation of the small heat shock proteins IpoA/B (63), similar to Cd(II), which also damages FeS-clusters (22, 64).

In summary, Cu(II) is reduced to Cu(I) in the periplasm, under anoxic conditions more rapidly than under oxic conditions, and Cu(I) is a better substrate for import into the cytoplasm than Cu(II). Copper damages by formation of hydroxyl radicals in the periplasm, and in the cytoplasm by acting on FeS cluster or preventing their assembly, and by binding to and unfolding proteins. Consequently, cytoplasmic and periplasmic functions are needed to protect either compartment against copper-mediated damage, and the cytoplasmic membrane between them. Tolerance functions should decrease the availability of Cu(I), protect or repair targets that are sensitive to Cu(I)-mediated damage.

### The strongest one is not strong enough alone: Cup needs partners.

*C. metallidurans* CH34 wild type contains four P_IB1_-type ATPases able to export Cu(I) from the cytoplasm to the periplasm. CupA and the plasmid pMOL30-encoded CopF are part of copper resistance determinants and only *cupA* is present in the plasmid-free strain AE104 that was used in this study. The *cupA* gene is followed by the gene for the metal-binding MerR-type and CueR-orthologous regulator *cupR* (49, 65) downstream of *cupA*. The gene *cupC* for a copper chaperone (29, 30) is located on the other DNA strand (Fig. S1C). CtpA and RdxI are “anabolic” P_IB1_-type ATPases involved in the assembly of periplasmic copper sites of copper-dependent proteins (4, 13, 66). The contribution of all four proteins to copper homeostasis of *C. metallidurans* CH34 wild type and its plasmid-free derivative AE104 has been characterized (8); however, the IC_50_ values published there and in this study cannot be directly compared because the Tris-buffered mineral salts medium has been improved in the meantime to reveal effects of metal starvation conditions at higher resolution. This has been accomplished by using mineral salts of higher purity. Especially, the sodium sulfate source previously used contained a larger amount of iron and zinc contaminations. This protected the cells so that the IC_50_ value of AE104 was 956 μM (8) compared to 615 μM published here (Table 1), and the IC_50_ of the Δ*cupA* mutant of strain AE104 was 400 μM (8) compared to 13.5 μM (Table 1), respectively. In strain AE104 cultivated in the previously used version of TMM, additional deletion of *rdxI* or of *ctpA* in the Δ*cup* strain decreased the IC_50_ value by half and deletion of both down to 25% of the IC_50_ of the Δ*cup* mutant (8). This would be an effect like a deletion of *cus* or *cop* in the Δ*cup* mutant (Table 1).

CupA is the only P_IB1_-type ATPase in strain AE104 that is upregulated by excess cytoplasmic Cu(I) ions via the CueR-orthologue CupR and accepts its substrate from the cytoplasmic copper chaperone CupC (29, 30, 49, 65). Deletion of the *cupC/AR* determinant (Fig. S1D) results in a strong decrease of copper resistance in all mutant backgrounds (Table 1, Table S1). The Δ*cup* deletion decreased the resistance level of strain AE104 45.6-fold to a resistance level half of that of a Cup^+^ quadruple mutant deleted in all the other copper resistance determinants (Δ*cop Δcus Δgig ΔgshA*, 29.0 μM versus 13.5 μM, Table 1). Consequently, the Cup system is the strongest contributor to copper resistance in *C. metallidurans* because it mediates efflux of excess cytoplasmic Cu(I) to the periplasm, but the other four factors are needed for an additional 21-fold increase in copper resistance. Efflux and influx systems adjust the concentration of their substrate in a cellular compartment via a kinetic flow equilibrium of the export and import rates (67, 68). This explains the necessity of interaction partners for CupA, which influence import of copper into the cytoplasm.

### The kinetic flow equilibrium of Cop and Cup controls the cytoplasmic copper levels.

The chromid-encoded *cop* determinant of the plasmid-free strain AE104 (Fig. S1E) contains genes for a periplasmic Cu(I) and Au(I) oxidase CopA (11), which is an orthologue of CueO from E. coli (16, 17, 69, 70). Additional *cop* products are the possible outer membrane-attached protein CopB, the periplasmic protein CopC, and the inner membrane protein CopD (71). Expression of *cop* in nonamended TMM medium is below the threshold of nucleotide activities per kilobase of exon model per million mapped reads (NPKM) = 10 (Fig. S1E) but expression can be upregulated about 10-fold by metal stress. The two-component regulatory system CopRS, which is encoded adjacent to *copABCD* on the other DNA strand, should be responsible for this upregulation.

CopC-like proteins usually contain distinct Cu(I) and Cu(II) binding sites and they are frequently fused as domain to a CopD-like domain (72) as in Bacillus subtilis (73) to form another copper uptake system, which is independent of the three import routes outlined above. Since CueO and its relatives are copper-dependent proteins, they are transported by the twin-arginine transport (TAT) system in a partially folded state (74). Cytoplasmic copper ions are needed to fold CopA/PcoA/CueO-like proteins and export them into the periplasm (71). It would be a disaster to the cells if a CopCD copper uptake system was produced in response to high periplasmic copper concentrations and would subsequently increase copper import into the cytoplasm and increase copper-mediated damage. Copper imported by CopCD should be exclusively used to fold CopA and not be released into the cytoplasm.

The outer membrane-attached and metal-binding (75) CopB protein contains at least two metal-binding motifs, HXHXCHXXH and EHXXXHXXDEH, which resemble His-rich Cu(II)-binding sites of CopC/PcoC-like proteins (76). CopA oxidizes Cu(I) back to Cu(II) which has two effects. First, CopA protects against copper-mediated killing (Table 2). Copper ions released from solid copper surfaces kill by damaging the cytoplasmic membrane (77–79). The cells are killed by the production of superoxide radicals by Cu(I)-mediated reduction of molecular oxygen, which is also responsible for the copper-mediated hydroxyl radical formation in the periplasm (54). Reoxidation of Cu(I) to Cu(II) in the periplasm by CopA prevents damage of the cytoplasmic membrane by reactive oxygen species and subsequent killing of the cells. CopB binds the resulting Cu(II) ions to prevent anew reduction. Second, since Cu(I) is a better substrate for copper uptake than Cu(II), oxidation of Cu(I) to Cu(II) by CopA decreases copper accumulation (Table 3). Acting together on the kinetic flow equilibrium that determines the cytoplasmic copper concentration, Cop and Cup increase the IC_50_ value of the respective Cop^+^ Cup^+^ triple deletion mutant Δ*cus Δgig ΔgshA* to 472 μM, 77% of the IC_50_ of the parent. On the other hand, Cop alone was not able to mediate a resistance level above the IC_50-min_ unless in cooperation with Cup, or Cus, GSH, or Gig (Table 1, Table S1). This indicates that Cus, Gig, and GSH should decrease the cytoplasmic or periplasmic Cu(I) concentration, repair Cu(I)-mediated membrane damage, or support Cop in another way.

### Cus exports periplasmic but not many cytoplasmic Cu(I) ions to the outside.

Reminiscent to *cop*, the *cusDCBAF* determinant on the chromid (Fig. S1C) is not expressed in nonamended mineral salts medium but upregulated under metal stress conditions. Only the *cusD* gene is expressed in nonamended medium. There is no two-component regulatory system able to sense copper encoded in the vicinity of *cus* so that CopRS might be responsible for *cus* expression like CusRS from E. coli, which also controls expression of *pcoE* (80). CusD is a periplasmic lipoprotein with a TAT leader as indicated by a signalP prediction (81) (Fig. S5) with several metal binding motifs such as MXMXXMDEHXXMEXXMXCXDM, CMXHC, HXDH, and HXXXHCC (68), indicating the potential to sequester Cu(I). Periplasmic CusF-like proteins were first found in E. coli (19) and bind Cu(I) to a WXHD-MXM site (82, 83). CusF can accept copper ions from the CupA-orthologue of E. coli (84) and deliver them to the CusCBA transenvelope efflux complex (85, 86). The CusF orthologue from *C. metallidurans* is only distantly related to the E. coli protein although both belong to the CusF-Ec protein superfamily. The copper binding site of the E. coli protein is not present in the *C. metallidurans* protein but Met-rich stretches that might represent three triple-Met Cu(I) binding sites, which would allow CusF from *C. metallidurans* to accept Cu(I) from CusD.

The CusCBA transenvelope efflux complex was first identified in E. coli (18, 19, 80). Because CusCBA can substitute a missing periplasmic CueO/CopA/PcoA-type Cu(I) oxidase (16, 70) and the periplasmic CusF delivers copper ions directly to this protein complex (85, 86), CusCBA exports periplasmic Cu(I) to the outside. In *C. metallidurans*, *cus* expression is strongly upregulated in a *cop* strain (Fig. 2A), which is unable to oxidize periplasmic Cu(I), so that periplasmic Cu(I) regulates *cus* expression. This may be accomplished by the CopRS two-component regulatory system, which is still present in *Δcop* mutant cells (Fig. S1E). The *gig* determinant is also upregulated in *Δcop* mutants (Fig. 2C), so that periplasmic Cu(I) also serves as inducer for *gig* expression. Metal-sensing histidine kinases such as CopS and CusS indeed sense periplasmic copper ions (9) so that regulation of *cus* expression in *C. metallidurans* by periplasmic copper ions is highly probable. Since the *gig* operon was more strongly upregulated in a Δ*cus Δcop* double mutant than in a Δ*cop* single mutant (Fig. 2C), presence of Cus clearly decreases the periplasmic Cu(I) concentration in *C. metallidurans*.

*C. metallidurans* can survive under anaerobic conditions by nitrate respiration (51) but the oxygen-dependent CopA protein cannot oxidize Cu(I) here; CusCBA would be important to remove periplasmic Cu(I) especially under anaerobic conditions, as has been shown for E. coli (17, 87). This makes Cus- together with Cup-like systems into the most frequently occurring copper resistance systems in gamma proteobacteria (88) and in natural communities (36).

As previously shown for another metal-exporting efflux system (89), CusA can export *in vitro* metal ions across a membrane that would resemble the cytoplasmic membrane *in vivo* (90, 91). Presence of an open periplasmic and a cytoplasmic copper binding site and a transport channel between them, however, would result in a copper uniport from the periplasm to the cytoplasm. The CusA-mediated transport of Cu(I) from the cytoplasm across the membrane has either no relevance *in vivo* or a regulatory function, for instance, CusCBA functions only when sufficient cytoplasmic copper ions are present. In *C. metallidurans*, Cus alone was not able to mount any copper resistance above the IC_50-min_ value (Table 1, Table S1). Presence of Cus increased copper resistance only in the presence of Cup, Cop, or Gig plus GSH. Export of excess cytoplasmic Cu(I) by CupA to the periplasm and successively by CusF and CusCBA to the outside would sufficiently explain the interaction of Cup and Cus, without assuming an export of cytoplasmic Cu(I) by Cus. Periplasmic Cu(I) ions oxidized by CopA back to Cu(II) may result in a new reduction of the ions not sequestered by CopB, which would be a futile cycle and export of periplasmic Cu(I) by Cus could interrupt this cycle. This function of Cus also fully explains the fact that Cus can substitute a missing Cop system in Cup^+^ strains. On the other hand, together with Cop, Gig, and GSH, Cus mediates the copper resistance level of the *Δcup* mutant of IC_50_ = 13.5 μM. This IC_50_ is 3.17-fold decreased by a Δ*cus* deletion (Table S1). Cus was also able to substitute missing Cup in Cop^+^ strains, and Cop as well as Cup were able to substitute a missing Cus, despite the different functions of Cup and Cop.

Together with Gig and GSH, Cus was able to increase copper resistance of *C. metallidurans* 4.2-fold above the IC_50-min_ value. The Cop system needs either Gig or GSH to mediate an 8.9- or 4.7-fold increase, respectively, above the IC_50-min_ value but Cus requires them both to substitute 1/3 of the performance of Cop. Gig and GSH perform an overlapping function with respect to Cop but an essential, additive function with respect to Cus. Possible contributions of the Gig and GSH to copper tolerance could be (i) repair of copper-mediated damage, (ii) oxidation of periplasmic Cu(I) to substitute Cop, (iii) sequestration of cytoplasmic or periplasmic copper ions, or (iv) reduction of Cu(II) to Cu(I) to feed the resulting ion into Cus-mediated efflux from the periplasm or the cytoplasm. A missing Cop system resulted in increased accumulation of copper by the cells even in the presence of Cup because more periplasmic Cu(I) as the substrate for import into the cytoplasm was available and the kinetic flow equilibrium of uptake and CupA-mediated export reactions reached a new, higher concentration level. GSH quenched copper accumulation in Δ*cop* cells by interacting with Cus. Gig could substitute GSH here to some part (Table 3). Repair of copper-mediated damage by Gig or GSH would not explain this fact. Sequestration of copper by Gig or GSH would increase the copper content of *Δcop* cells independent of Cus, leaving a redox change of copper ions in the periplasm or cytoplasm as possible functions of Gig and GSH.

The redox potential of Cu(II)/Cu(I) of E_0_’ = −267 mV (40) is close to the redox potential of GSSG/GSH of E_0_’ = −240 mV under standard conditions and E_0_’ = −260 mV *in vivo* (92, 93), leading to a ratio of GSH to GSSG in the cytoplasm of 1,000:1, according to the Nernst equation (22). Taking the low concentration of copper in the cytoplasm and the high ratio GSH to GSSG into account, oxidation of Cu(I) by GSSG is not a favorable reaction but instead Cu(II) is rapidly reduced by GSH (26–28, 94, 95). The resulting Cu(I) is promptly bound by CupC and exported by CupA. In the periplasm and in the Δ*cop* strain with its upregulated *cus* expression, a reduction of Cu(II) to Cu(I) for the purpose of exporting the resulting Cu(I) by CusCBA so that Cu(I) cannot be imported into the cytoplasm would require an efficient Cu(I) removal by CusCBA. The increased copper sensitivity of E. coli under anaerobic conditions (10) would not agree with such an efficient Cu(I) removal by Cus, at least not in E. coli.

In the periplasm, the DsbA/DsbB-, thioredoxin/DsbB-, or glutaredoxin/GSSG-mediated higher redox level (96) could indeed oxidate Cu(I) back to Cu(II) as an alternative, CopA-independent pathway of Cu(I) oxidation, which cooperates with removal of periplasmic Cu(I) by Cus. Adding Gig plus GSH to a Cus^+^-only quadruple mutant increased its copper resistance 33% compared to the increase resulting from the addition of Cop to this quadruple mutant (Table S1), so that the efficiency of a possible Gig/GSSG electron shuttle should be 33% of that of CopA, and both Gig and GSSG are needed for this little increase in resistance. The decrease of the GSH content of the *C. metallidurans* Δ*cus* mutant (Table 4) would agree to this fact because more exported, periplasmic GSSG would be needed if Cus no longer exports periplasmic Cu(I) ions.

On the other hand, Gig or GSH are required for CopA to mediate any increase in the resistance level. Since CopA is 3-times more efficient than Gig/GSSG, both are not needed for Cu(I) oxidation when CopA is present. But CopA is exported in a partially folded form (74). GSH may be required for efficient loading of copper into nascent CopA in the cytoplasm and Gig in the periplasm. This would explain the 4.72-fold increase above the IC_50-min_ value resulting from addition of Cop to the Gig^+^-only and the 9.81-fold increase by addition of Cop to the GSH-only quadruple mutants, respectively (Table S1).

These contributions of GSH and Gig to copper tolerance also indicate that the main physiological function of Cus is export of periplasmic Cu(I) to the outside. If Cus also exports cytoplasmic Cu(I) ions, the export rate should be in the range of Cu(I) export by the anabolic P_IB1_-type ATPases RdxI and CtpA and much lower than that mediated by CupA. Cus interacts with Cup to form an export route of excess cytoplasmic Cu(I) by CupA-CusF-CusCBA, with Cop to remove periplasmic Cu(I) by oxidation, and with Gig plus GSH to the same end.

### Hints concerning a possible function of Gig.

The *gigPABT* operon (Fig. 3) is in a divergon situation with the gene for the extracytoplasmic function (ECF) sigma factor (97–102) gene *rpoQ* and a putative gene for an antisigma factor. In nonamended growth medium, *gig* is expressed on a low level around the threshold of NPKM = 10 (Fig. S1A). Due to the low expression level, no promoter upstream of *gig* has been identified (103). The *rpoQ-rsqA* region for the sigma factor and its predicted membrane-bound antisigma factor RsqA depends on the main housekeeping sigma factor RpoD (103). No evidence for initiation of expression of *gig* from a RpoQ-dependent RNA polymerase has been obtained since deletion of *rpoQ* results in an upregulation of *gig* expression in metal-treated mutant cells (100) due to a compensatory mechanism of the sigma factor network of *C. metallidurans*. Since *gig* was clearly upregulated in response to copper ions in Δ*cop* cells more than in parent cells and in Δ*cop Δcus* cells more than in Δ*cop* cells (Fig. 2B), *gig* is under the control of periplasmic signals. Regulation by RpoQ and its antisigma factor can be assumed despite the currently lacking further experimental evidence. This would also hint that Gig may protect against stress originating in the periplasm (98, 102).

The first Gig protein is GigP (Rmet_4685), a small putative periplasmic protein without an outstanding metal binding motif. The overall size is 94 aa in length of the preprotein, minus 22 aa for the leader as determined by SignalP 5.0. The remaining 72-aa peptide contains 4 Cys and 5 Met residues not very close to each other. The low hydrophobicity of the 72 aa argues for a periplasmic rather than an integral membrane protein. GigA (Rmet_4684) is a metal-binding protein with a few His-containing putative metal-binding motifs, which resemble trinuclear zinc-binding sites involved in phosphodiester cleavage in endonuclease IV involved in base excision repair (104). GigB (Rmet_4683) is a predicted cytoplasmic protein with a thioredoxin fold, His- and Cys-containing metal-binding motifs. GigB contains a DUF2063 domain at the amino terminus, which occurs in the DNA-binding protein NGO1945 from Neisseria gonorrhoeae (105). GigT (Rmet_4682) has three or four predicted transmembrane spans and is loosely related to DoxX (COG2259), a subunit of a terminal quinol oxidase in an archaeon. While GigA and GigB might be associated with the DNA, GigP and GigT may have a function in the cellular envelope, cytoplasmic membrane, or periplasm.

The distant relationship of GigT with a component of a quinol oxidase indicates the potential of GigT and GigP to oxidize periplasmic Cu(I)-GSSG and channel the resulting electron into the quinol pool, which would explain why Gig plus glutathione enable Cus to mediate some increase in copper resistance above the IC_50-min_ (Table 1, Table S1). Repair or protection of the DNA by GigB and GigA is also in agreement with the importance of Gig in the mutants Δ*cup ΔgshA*, *Δcup Δcus ΔgshA*, and Δ*cop Δcup*, which are devoid of Cup-mediated removal of cytoplasmic Cu(I) ions in combination with increased import and decreased removal of Cu(I) bound to proteins by GSH, including zinc-binding enzymes involved in DNA repair.

Cop needs Cup, Cus, GSH, or Gig to mediate some increase in copper resistance above the IC_50-min_ level. Cup is required as Cu(I)-exporting counterpart for excess cytoplasmic copper ions while Cop oxidizes periplasmic Cu(I) back to Cu(II) to decrease copper uptake into the cytoplasm. Cop and Cus remove periplasmic Cu(I) alternatively by oxidation or efflux, respectively. GSH may be required to load copper imported by CopCD in the cytoplasm into the pre-CopA apoprotein ahead of its TAT-mediated export to the periplasm. GigPT could also be involved of an efficient activation of apo-CopA, e.g., by mobilization of periplasmic Cu(I) for subsequent import by CopCD. In summary, Gig contributes to copper tolerance, but its contribution is small, only visible in a few mutant backgrounds and the biochemical mechanisms behind its function have not been revealed yet (Fig. 1). This may change when gold complexes are added to the picture.

### GSH does not form a copper-complexing pool in the cytoplasm.

In Streptococcus pyogenes, glutathione buffers excess intracellular copper (106). The higher copper content of GSH^+^ cells of *C. metallidurans* compared to Δ*gshA* cells indicates that presence of GSH also leads to increased cellular copper binding in this bacterium (Table 3) but presence of GSH did not increase the copper resistance of the Cup^+^-only mutant (*Δcop Δcus Δgig ΔgshA*) or Δ*cup* mutant at all (Table S1). GSH was not required for function of Cup and did not substitute a missing Cup system.

In the presence of copper ions, copper-catalyzed oxidation of GSH to GSSG produces hydrogen peroxide and superoxide radicals (27, 95). Cu(II) is immediately reduced by the high intracellular GSH content to Cu(I)-GSH_2_ complexes, which react with molecular oxygen to Cu(II)-GSSG and superoxide radicals (95). GSH is able to reduce Cu(II) bound to GSSG again and this reaction is quantitative and complete due to the high intracellular concentrations of GSH (26). This is an exceptional reaction only catalyzed by GSH while other thiols bind Cu(II) and convert subsequently to time-stable Cu(I)-thiol complexes. Consequently, GSH cannot protect the cytoplasm against copper-mediated oxidative damage but is the source of it in aerobic bacteria. Cytoplasmic copper chaperones such as CupC in *C. metallidurans* or its orthologue from E. coli (107, 108) are needed to keep Cu(I) away from GSH and to deliver the ion to CupA-like proteins for export to the outside. One function of GSH is to prevent interaction of copper with cytoplasmic proteins, which leads to unfolding reactions (109), a second to repair oxidative damage caused by its own interaction with Cu(I).

GSH in *C. metallidurans* was not required for the function of CupA but nearly all mutants with a Δ*cop* mutation now needed GSH, except those with a Δ*cop Δgig* double mutation. Both, Cu(I) and Cu(II) bind to GSSG, the oxidized form of GSH that is the predominant form in the periplasm (26, 95, 96, 110). In the cytoplasm, only GSH is able to reduce Cu(II) bound to GSSG (26). In the periplasm, a Gig-mediated oxidation of Cu(I)-GSSG would explain why GSH is especially required in *Δcop* mutants (Table 1) for removal of periplasmic Cu(I) ions as outlined above. GSH can be exported into the periplasm by a CydDC-type bacterial glutathione exporter (111). Four proteins in *C. metallidurans* are related to CydD: Rmet_0391 or AtmA (29% identity in 428 aa), Rmet_0705 (27% identity in 515 aa), Rmet_2516 (30% identity in 426 aa), and Rmet_0757 (24% identity in 493 aa). Three proteins are related over a long range to CydC: Rmet_2516 (37% in 289 aa), Rmet_0705 (28% in 493 aa), and Rmet_0757 (26% in 508 aa) again. Rmet_0757 is probably the lipid A exporter MsbA but AtmA, Rmet_0705, and Rmet_2516 could export GSH or GSH complexes, and AtmA is involved in nickel tolerance in *C. metallidurans* (112).

### Conclusion.

Copper resistance in the plasmid-free *C. metallidurans* strain AE104 is accomplished by the interaction of many systems and not predominantly by the function of a single factor (Fig. 1). The Cup system is under the control of the cytoplasmic Cu(I) content via the MerR-type regulator CupR, binds these ions to CupC, and exports them to the periplasm by the P_IB1_-type ATPase CupA. In analogy to other bacteria, Cu(I) may be forwarded to CusCBA for further export to the outside by CusF. While the CusCBA transenvelope efflux system clearly affects the periplasmic copper concentration, no evidence argues for an efficient CusCBA-mediated efflux of cytoplasmic Cu(I) ions *in vivo*. In contrast to E. coli, the Cus system from *C. metallidurans* additionally contains the periplasmic TAT-exported lipoprotein CusD, which contains a copper binding site, may sequester periplasmic Cu(I) close to the cytoplasmic membrane, and forward the ions via CusF to CusCBA for export. While Cus removes excess periplasmic Cu(I) by export to the outside, the Cop system oxidizes Cu(I) to Cu(II), which is inferior to Cu(I) as the substrate for copper import, leading to a Cop-mediated decreased copper accumulation. Moreover, Cop also protects against copper-mediated oxidative damage and subsequent killing of the cells. CopA is a copper-containing oxygen-dependent Cu(I) oxidase. CopC and CopD may import copper into the cytoplasm for loading of copper into CopA and subsequent TAT-mediated export into the periplasm. Glutathione could stimulate this process. CopB is attached to the outer membrane and seems to bind Cu(II) to prevent reduction of Cu(II) to Cu(I) by respiratory chain components in the cytoplasmic membrane. Glutathione seems not to be a cytoplasmic Cu(I) buffer because interaction of GSH with copper ions leads to production of superoxide radicals. Instead, it is required for full function of Cop, maybe by assisting insertion of copper into apo-CopA, and periplasmic GSSG may interact with the Gig system to mediate a CopA-independent oxidation of periplasmic Cu(I). Moreover, Gig, which is under the control of periplasmic copper ions and a minor contributor to copper tolerance, contains two proteins that may protect the DNA against copper-mediated damage in the absence of CupA and GSH, or repair this damage, or circumvent DNA-interacting zinc-dependent proteins inactivated by copper. In comparison with the plasmid-free *C. metallidurans* strain AE104, the wild type additionally contains a huge copper resistance region on plasmid pMOL30, which adds another level of sophistication to copper homeostasis of this bacterium.

## MATERIALS AND METHODS

### Bacterial strains and growth conditions.

Strains used for experiments were derivatives of the plasmid-free derivative AE104 of *C. metallidurans* CH34 (3) and are listed in Table S5 in the supplemental material. Tris-buffered mineral salts medium (3) containing 2 g sodium gluconate/l (TMM) was used to cultivate these strains aerobically with shaking at 30°C. The medium had been improved compared to the previously published medium by choosing mineral salts with a higher purity. Solid Tris-buffered media contained 20 g/L agar. Strains were routinely transferred to fresh TMM plates every 2 weeks and taken from the −80°C stock culture twice a year.

### Dose-response growth curves in 96-well plates were conducted in TMM.

A preculture was incubated at 30°C, 200 rpm up to early stationary phase, then diluted 1:20 into fresh medium and incubated for 24 h at 30°C and 200 rpm. Overnight cultures were used to inoculate parallel cultures with increasing metal concentrations in 96-well plates (Greiner). Cells were cultivated for 20 h at 30°C and 1,300 rpm in a neoLab Shaker DTS-2 (neoLab, Heidelberg, Germany) and the optical density was determined at 600 nm as indicated in a TECAN Infinite 200 PRO reader (Tecan Group Ltd., Männedorf, Switzerland). To calculate the IC_50_ values (inhibitory concentration: metal concentration that led to turbidity reduction by half) and the corresponding *b* value (measure of the slope of the sigmoidal dose-response curve), the data were adapted to the formula OD(*c*) = OD0/{1 + exp([c − IC_50_]/*b*)}, which is a simplified version of a Hill-type equation, as introduced by Pace and Scholtz (1997) (113, 114). OD(*c*) is the turbidity at a given metal concentration, OD0 that had no added metal and *c*, the metal concentration.

### β-galactosidase assay.

*C. metallidurans* cells with a *lacZ* reporter gene fusion were cultivated as a preculture in TMM containing 1.5 g/L^−1^ kanamycin at 30°C, 250 rpm for 30 h, diluted 20-fold into fresh medium with 1 g/L^−1^ kanamycin, incubated with shaking at 30°C for 24 h, diluted 50-fold into fresh medium, and incubated with shaking at 30°C until a cell density of 100 Klett units was reached. This culture was distributed into sterile 96-well plates (Greiner Bio-One, Frickenhausen, Germany). After addition of metal salts, incubation in the 96-well plates was continued for 3 h at 30°C in a neoLab Shaker DTS-2 (neoLab Migge Laborbedarf, Heidelberg, Germany). The turbidity at 600 nm was determined in a TECAN Infinite 200 Pro reader (TECAN, Männedorf, Switzerland) and the cells sedimented by centrifugation at 4°C for 30 min at 4,500 × *g*. The supernatant was discarded and the cell pellets were frozen at −20°C. For the enzyme assay, the pellet was suspended in 190 μL Z buffer (60 mM Na_2_HPO_4_, 40 mM NaH_2_PO_4_, 10 mM KCl, 1 mM MgSO_4_, 0.5 M beta-mercaptoethanol) and 10 μL permeabilization buffer was added (6.9 mM CTAB, cetyl-trimethyl-ammonium bromide, 12 mM sodium deoxycholate). The suspension was incubated with shaking at 30°C and 20 μL ONPG solution (13.3 mM ortho-nitrophenyl-beta-d-galactopyranoside in Z-buffer without beta-mercaptoethanol) were added. Incubation was continued with shaking in a neoLab Shaker DTS-2 at 30°C until the yellow color of *o*-nitrophenol was clearly visible and stopped by addition of 50 μL 1 M Na_2_CO_3_. The extinction at 420 nm and 550 nm was measured in a TECAN Infinite 200 Pro reader. The activity was determined as published (115) with a factor of 315.8 μM calculated from the path length of the 96-well plate and the extinction coefficient of *o*-nitrophenol:

$$\text{activity }=\text{ 315}.\text{8 }\mu \text{M }*\;\left\{{\text{E}}_{\text{42}0}\;-\;\left(\text{1}.\text{75 }*{\text{ E}}_{\text{55}0}\right)\right\}/\text{reaction time}$$

specific activity: activity divided by the cellular dry mass as published (115).

### Genetic techniques.

Standard molecular genetic techniques were used (116, 117). For conjugative gene transfer, overnight cultures of donor strain E. coli S17/1 (118) and of the *C. metallidurans* recipient strains grown at 30°C in Tris- buffered medium were mixed (1:1) and plated onto nutrient broth agar. After 2 d, the bacteria were suspended in TMM, diluted, and plated onto selective media as previously described (116). Primer sequences are provided in Table S6.

### Gene deletions.

Primer sequences are also provided in Table S6. Plasmid pECD1002, a derivate of plasmid pCM184 (119), was used to construct deletion mutants. These plasmids harbor a kanamycin resistance cassette flanked by *loxP* recognition sites. Plasmid pECD1002 additionally carries alterations of 5 bp at each *loxP*-site. Using these mutant *lox* sequences, multiple gene deletions within the same genome are possible without interferences by secondary recombination events (120, 121). Fragments of 300 bp upstream and downstream of the target gene were amplified by PCR, cloned into vector pGEM T-Easy (Promega), sequenced, and further cloned into plasmid pECD1002. The resulting plasmids were used in a double-crossover recombination in *C. metallidurans* strains to replace the respective target gene by the kanamycin-resistance cassette, which was subsequently also deleted by transient introduction of *cre* expression plasmid pCM157 (119). Cre recombinase is a site-specific recombinase from the phage P1 that catalyzes the *in vivo* excision of the kanamycin resistance cassette at the *loxP* recognition sites. The correct deletions of the respective transporter genes were verified by Southern DNA-DNA hybridization. For construction of multiple deletion strains, these steps were repeated. The resulting mutants carried a small open reading frame instead of the wild-type gene to prevent polar effects.

### Gene insertions and disruptions.

For reporter operon fusions, *lacZ* was inserted downstream of several targets. This was done without interrupting any open reading frame downstream of the target genes to prevent polar effects. The 300- to 400-bp 3′ ends of the respective target genes were amplified by PCR from total DNA of strain AE104 and the resulting fragments cloned into plasmid pECD794 (pLO2-*lacZ*) (122). The respective operon fusion vectors (pECD1386 for *cusF-lacZ*, pECD1667 for *gigT-lacZ*) were inserted into the open reading frame of the target gene by single crossover recombination. Using the 300-bp 5′ part of the *gshA* gene, this procedure was also used to interrupt the *gshA* gene with pECD1668 in strain AE104 parent strain and its mutant derivatives.

### PCR.

DNA was amplified by PCR with 0.2 μM each primer and 1.5 U of *Taq* polymerase (Roche Diagnostics GmbH, Mannheim, Germany). All primer pairs used are listed in Table S6. The resulting amplified DNA was separated on agarose gels stained with ethidium bromide (117).

### RNA isolation.

*C. metallidurans* cells were cultivated as described above. At a cell turbidity of 150 Klett, metal salts were added. After a 10- or 30-min incubation at 30°C, the cells were rapidly harvested and stored at −80°C. Total RNA was isolated as published (123) DNase treatment was performed. RNA concentration was determined photometrically, and RNA quality was checked on formamide gels (117).

### RT-PCR.

For the RT reaction, 1 μg of total RNA and 0.1 μg hexamer primers were incubated at 65°C for 5 min and snap-cooled on ice. After addition of 0.5 mM (each) dATP, dGTP, dTTP, and dCTP, 20 mM DTT, and 100 U of reverse transcriptase (superscript II) in reaction buffer (Thermo Fisher Scientific, Germany), reverse transcription proceeded for 10 min at room temperature, followed by 1 h at 42°C. After finishing the RT reaction, the enzyme was inactivated at 70°C for 10 min. Then, 1 μL of the resulting cDNA was amplified by PCR with 0.2 μM each primer and 1 U of Taq-Polymerase (Roche Diagnostics GmbH, Mannheim, Germany). A no-template control and a no-RT (negative) control were performed under identical conditions as for the target genes.

### Live/Dead staining of surface-exposed cells.

Live/Dead staining (Live/Dead BacLight bacterial viability kit, LifeTechnologies, Darmstadt, Germany) differentiates between undamaged and damaged bacterial membranes by employing two fluorescence dyes, which intercalate into DNA. Live, undamaged bacteria fluoresce green, while membrane-damaged bacteria fluoresce red. This is because the green stain SYTO 9 penetrates the membrane of both undamaged and damaged cells, while the red stain propidium iodide can only penetrate damaged membranes, and after intercalating into the bacterial DNA, reduces the green fluorescence of SYTO 9.

A preculture incubated at 30°C, 200 rpm for 18 h in TMM (with kanamycin for mutant strains carrying a gene disruption) was diluted into fresh medium at 5% and incubated at 30°C shaking, 200 rpm for 24 h. Cells were diluted 10-fold into fresh medium and continue shaking at 200 rpm for 20 h. Cultures contained no copper or copper resembling the IC_50_ concentration (calculated from dose response experiments) unique for each strain. After incubation cells were harvested and washed two times in saline (0.85% NaCl solution). Pellets were resuspended in 80 μL saline and divided in two samples each containing 40 μL of suspension. One sample was diluted with 800 μL of saline (Live sample), the other with 70% isopropanol (Dead sample) and incubated for 1 h at room temperature. Cells were harvested, washed two times in saline and resuspended in 400 μL saline. Cell suspensions were mixed 1:1 in a 1:1 mixture of Syto9 and propidium iodide solution (L13152 Live/Dead BacLight bacterial viability kit, Thermo Fisher Scientific, Dreiech), incubated for 10 to 15 min and examined in a confocal fluorescence microscope (Carl Zeiss MicroImagine, Jena, Germany) with λ_Ex_ 450 to 490/546 nm, λ_Em_ 520/590 nm.

### Inductively coupled plasma mass spectrometry.

Cells were incubated in TMM for 20 h at 30°C shaking at 200 rpm, diluted 20-fold into fresh TMM medium, and shaking continued at 30°C for 24 h. Cells were diluted 50-fold into fresh medium containing added copper or not, and continued shaking at 30°C at 200 rpm until 150 Klett were reached (midexponential phase of growth). Then, 10 mL of the cells were harvested by centrifugation, washed twice with 50 mM TrisHCl buffer (pH 7.0) containing 50 mM EDTA at 0°C and suspended in 50 mM TrisHCl buffer (pH 7.0). For inductively coupled plasma mass spectrometry (ICP-MS) analysis, HNO_3_ (trace metal grade; Normatom/PROLABO) was added to the samples to a final concentration of 67% (wt/vol) and the mixture mineralized at 70°C for 2 h. Samples were diluted to a final concentration of 2% (wt/vol) nitric acid. Indium and germanium were added as internal standards at a final concentration of 10 ppb each. Elemental analysis was performed via ICP-MS using Cetac ASX-560 sampler (Teledyne, Cetac Technologies, Omaha, Nebraska), a MicroFlow PFA-200 nebulizer (Elemental Scientific, Mainz, Germany) and an ICAP-TQ ICP-MS instrument (Thermo Fisher Scientific, Bremen) operating with a collision cell and flow rates of 4.8 mL × min^−1^ of He/H_2_ (93%/7% [124]), with an Ar carrier flow rate of 0.76 L × min^−1^ and an Ar make-up flow rate at 15 L × min^−1^. An external calibration curve was recorded with ICP-multi-element standard solution XVI (Merck) in 2% (vol/vol) nitric acid. The sample was introduced via a peristaltic pump and analyzed for its metal content. For blank measurement and quality/quantity thresholds, calculations based on DIN32645 TMM were used. The results were calculated from the ppb data as atoms per cell as described (125).

### Glutathione determination.

Cells were cultivated as described above for ICP-MS. Next, 5 mg cells were harvested (15 min, 4,500 × *g*, 4°C) and washed twice with TMM. Cell pellets were resuspended in 100 μL 5% sulfosalicylic acid (SSA) solution and disrupted by three freeze-thawing cycles (liquid nitrogen, water bath at 37°C, 2 min per treatment). Cell debris was removed by centrifugation (10 min, 15,300 × *g*, 4°C). The supernatant was used to determine the protein concentration with the QuantiPro bicinchoninic acid assay (BCA assay) kit (Sigma-Aldrich, Taufkirchen, Germany) using bovine serum albumin as a standard and to measure the GSH content by the glutathione assay kit (CS0260, Sigma-Aldrich, Taufkirchen, Germany) according to manufacturer’s instructions. The enzymatic determination of the total amount of glutathione (GSH and GSSG) after deproteinization with SSA were measured photometrically at 412 nm by increasing amounts of TNB [5,5=dithiobis(2-nitrobenzoic acid)] using a kinetic assay.

### Statistics.

Student’s *t* test was used but in most cases the distance (D) value has been used several times previously for such analyses (7, 33, 34). It is a simple, more useful value than Student's *t* test because nonintersecting deviation bars of two values (D > 1) for three repeats always means a statistically relevant (≥95%) difference provided the deviations are within a similar range. At *n* = 4, significance is ≥97.5%, at *n* = 5 ≥ 99% (significant), and at *n* = 8 ≥ 99.9% (highly significant).
