# Supplementary material for: Full Copper Resistance in Cupriavidus metallidurans Requires the Interplay of Many Resistance Systems
Source: Appl Environ Microbiol. 2023 May 16;89(6):e00567-23. doi: 10.1128/aem.00567-23 (PMC10304878; doi:10.1128/aem.00567-23)
Supplement: Supplemental file 1 — Supplemental material. Download aem.00567-23-s0001.pdf, PDF file, 5.6 MB [file aem.00567-23-s0001.pdf]

Supplementary Table S1. Effect of deletions in copper resistance genes[a]

Bacterial parent strain	IC_{50}, (µM)	Bacterial mutant strain	IC_{50}, (µM)	Q	D
Deletion of *cup*					
Δcus	743±62	*Δcup Δcus*	4.25±0.89	**175**	11.8
AE104	615±68	*Δcup*	13.5±1.1	**45.6**	8.70
Δcus ΔgshA	612±32	*Δcup Δcus ΔgshA*	3.83±1.37	**160**	18.0
Δcus Δgig	597±26	*Δcup Δcus Δgig*	2.22±0.14	**269**	23.1
Δgig	580±30	*Δcup Δgig*	11.00±2.11	**52.7**	17.8
Δcop Δgig	492±50	*Δcop Δcup Δgig*	0.43±0.21	**1145**	9.73
Δcus Δgig ΔgshA	472±49	*Δcup Δcus Δgig ΔgshA*	0.51±0.12	**925**	9.65
Δgig ΔgshA	458±43	*Δcup Δgig ΔgshA*	6.22±1.03	**73.7**	10.3
ΔgshA	450±6	*Δcup ΔgshA*	13.2±2.5	**34.0**	48.8
Δcop	389±43	*Δcop Δcup*	3.41±0.62	**114**	8.79
Δcop Δcus	197±21	*Δcop Δcup Δcus*	0.81±0.23	**243**	9.30
Δcop ΔgshA	161±24	*Δcop Δcup ΔgshA*	0.42±0.10	**383**	6.56
Δcop Δcus Δgig	158±16	*Δcop Δcup Δcus Δgig*	0.47±0.10	**336**	9.98
Δcop Δgig ΔgshA	110±9	*Δcop Δcup Δgig ΔgshA*	0.46±0.07	**240**	11.6
Δcop Δcus Δgig ΔgshA	29.0±4.4	*Δcop Δcup Δcus Δgig ΔgshA*	0.44±0.16	**65.9**	6.22
Δcop Δcus ΔgshA	26.4±2.9	*Δcop Δcup Δcus ΔgshA*	0.43±0.09	**61.5**	8.72
Deletion of *cop*					
Δcus	743±62	*Δcop Δcus*	197±21	**3.77**	6.61
AE104	615±68	*Δcop*	389±43	1.58	2.04
Δcus ΔgshA	612±32	*Δcop Δcus ΔgshA*	26.4±2.9	**23.1**	16.6
Δcus Δgig	597±26	*Δcop Δcus Δgig*	158±16	**3.78**	10.6
Δgig	580±30	*Δcop Δgig*	492±50	1.18	1.09
Δcus Δgig ΔgshA	472±49	*Δcop Δcus Δgig ΔgshA*	29.0±4.4	**16.3**	8.33
Δgig ΔgshA	458±43	*Δcop Δgig ΔgshA*	110±9	**4.15**	6.66
ΔgshA	450±6	*Δcop ΔgshA*	161±24	**2.80**	9.38
Δcup	13.5±1.2	*Δcop Δcup*	3.41±0.62	**3.95**	5.69
Δcup ΔgshA	13.2±2.5	*Δcop Δcup ΔgshA*	0.42±0.10	**31.5**	4.95
Δcup Δgig	11.0±2.1	*Δcop Δcup Δgig*	0.43±0.21	**25.6**	4.56
Δcup Δgig ΔgshA	6.22±1.03	*Δcop Δcup Δgig ΔgshA*	0.46±0.07	**13.5**	5.24
Δcup Δcus	4.25±0.89	*Δcop Δcup Δcus*	0.81±0.23	**5.25**	3.07
Δcup Δcus ΔgshA	3.83±1.37	*Δcop Δcup Δcus ΔgshA*	0.43±0.09	**8.91**	2.33
Δcup Δcus Δgig	2.22±0.14	*Δcop Δcup Δcus Δgig*	0.47±0.10	**4.72**	7.29
Δcup Δcus Δgig ΔgshA	0.51±0.12	*Δcop Δcup Δcus Δgig ΔgshA*	0.44±0.16	*1.16*	0.25

continued

Supplementary Table S1 continued

Bacterial strain	IC_{50}, (µM)	Bacterial strain	IC_{50}, (µM)	Q	D
Deletion of *cus*					
AE104	615±68	*Δcus*	743±62	*0.83*	0.99
Δgig	580±30	*Δcus Δgig*	597±26	*0.97*	0.31
Δcop Δgig	492±50	*Δcop Δcus Δgig*	158±16	**3.12**	5.07
Δgig ΔgshA	458±43	*Δcus Δgig ΔgshA*	472±49	*0.97*	0.15
ΔgshA	450±6	*Δcus ΔgshA*	612±32	0.74	4.17
Δcop	389±43	*Δcop Δcus*	197±21	1.97	2.99
Δcop ΔgshA	161±24	*Δcop Δcus ΔgshA*	26.4±2.9	**6.09**	4.94
Δcop Δgig ΔgshA	110±9	*Δcop Δcus Δgig ΔgshA*	29.0±4.4	**3.81**	5.90
Δcup	13.5±1.2	*Δcup Δcus*	4.25±0.89	**3.17**	4.52
Δcup ΔgshA	13.2±2.5	*Δcup Δcus ΔgshA*	3.83±1.37	**3.46**	2.44
Δcup Δgig	11.0±2.1	*Δcup Δcus Δgig*	2.22±0.14	**4.95**	3.90
Δcup Δgig ΔgshA	6.22±1.03	*Δcup Δcus Δgig ΔgshA*	0.51±0.12	**12.2**	4.97
Δcop Δcup	3.41±0.62	*Δcop Δcup Δcus*	0.81±0.23	**4.21**	3.06
Δcop Δcup Δgig ΔgshA	0.46±0.07	*Δcop Δcup Δcus Δgig ΔgshA*	0.44±0.16	*1.05*	0.09
Δcop Δcup Δgig	0.43±0.21	*Δcop Δcup Δcus Δgig*	0.47±0.10	*0.91*	0.13
Δcop Δcup ΔgshA	0.42±0.10	*Δcop Δcup Δcus ΔgshA*	0.43±0.09	*0.98*	0.05
Deletion of *gig*					
Δcus	743±62	*Δcus Δgig*	597±26	1.24	1.67
AE104	615±68	*Δgig*	580±30	*1.06*	0.36
Δcus ΔgshA	612±32	*Δcus Δgig ΔgshA*	472±49	1.30	1.72
ΔgshA	450±6	*Δgig ΔgshA*	458±43	*0.98*	0.17
Δcop	389±43	*Δcop Δgig*	492±50	0.79	1.11
Δcop Δcus	197±21	*Δcop Δcus Δgig*	158±16	1.25	1.07
Δcop ΔgshA	161±24	*Δcop Δgig ΔgshA*	110±9	1.46	1.50
Δcop Δcus ΔgshA	26.4±2.9	*Δcop Δcus Δgig ΔgshA*	29.0±4.4	*0.91*	0.35
Δcup	13.5±1.2	*Δcup Δgig*	11.0±2.1	*1.23*	0.76
Δcup ΔgshA	13.2±2.5	*Δcup Δgig ΔgshA*	6.22±1.03	**2.13**	1.99
Δcup Δcus	4.25±0.89	*Δcup Δcus Δgig*	2.22±0.14	1.91	1.97
Δcup Δcus ΔgshA	3.83±1.37	*Δcup Δcus Δgig ΔgshA*	0.51±0.12	**7.51**	2.23
Δcop Δcup	3.41±0.62	*Δcop Δcup Δgig*	0.43±0.21	**7.93**	3.59
Δcop Δcup Δcus	0.81±0.23	*Δcop Δcup Δcus Δgig*	0.47±0.10	1.72	1.03
Δcop Δcup Δcus ΔgshA	0.43±0.09	*Δcop Δcup Δcus Δgig ΔgshA*	0.44±0.16	*0.98*	0.04
Δcop Δcup ΔgshA	0.42±0.10	*Δcop Δcup Δgig ΔgshA*	0.46±0.07	*0.91*	0.24

[a]Copper resistance was determined in 96-well plates at 30°C in all mutants and is provided in Table 1. Here, for all mutations, these data were additionally sorted in decreasing order of the IC_{50} values of the respective strains with or without *cup* (top), *cop, cus* and *gig* (bottom), respectively. The Q values give the IC_{50} ratios plus and minus the particular gene, D the respective distance value. Q values in italics indicate no difference between the IC_{50} value (D < 1), bold-faced values Q > 2. Mutants with a Δ*gshA* deletion are on shaded fields.

Supplementary Table S2. Percentage of dead cells in cultures of *C. metallidurans* mutant cells[a].

Bacterial strain	% dead cells							
	Saline				Isopropanol			
	GshA+ no Cu	GshA+ plus Cu	Δ*gshA* no Cu	Δ*gshA* plus Cu	GshA+ no Cu	GshA+ plus Cu	Δ*gshA* no Cu	Δ*gshA* plus Cu
AE104	4.93±2.40	9.12±5.98	2.81±1.76	5.54±3.23	98±3	100±0	97±3	100±1
Δcop	2.90±1.58	9.67±5.66	2.74±3.04	4.69±3.28	99±1	100±0	96±3	99±1
Δcup	2.43±2.85	8.61±6.25	1.24±0.62	**4.91±2.79**	99±1	100±0	95±4	98±4
Δcus	2.81±2.72	15.9±16.7	1.32±0.58	**7.04±4.36**	99±1	99±1	97±2	100±1
Δgig	3.45±0.71	5.73±2.68	2.54±1.91	4.10±2.38	100±0	100±0	97±3	100±0
Δcop Δcup	3.96±2.04	10.5±4.9	9.47±5.69	9.37±3.62	100±0	99±1	98±2	98±5
Δcop Δcus	3.57±2.11	**27.3±17.1**	2.75±1.89	**13.7±5.6**	100±0	99±1	96±3	100±1
Δcop Δgig	2.51±2.30	10.1±10.1	4.50±0.39	**30.7±10.0**	100±0	100±0	98±1	99±2
Δcus Δgig	3.41±3.88	18.2±26.4	2.24±0.44	3.64±2.07	100±1	100±0	97±2	100±1
Δcup Δgig	4.53±2.20	4.66±4.53	4.35±1.41	**10.6±1.9**	100±1	100±1	95±3	99±3
Δcup Δcus	4.35±3.16	2.58±2.65	4.19±2.23	**20.0±13.5**	99±1	100±0	93±4	100±0
Δcup Δcus Δgig	5.12±2.44	3.78±2.42	3.95±3.57	2.76±2.84	100±0	100±1	97±2	99±2
Δcop Δcus Δgig	5.51±3.13	17.1±6.1	6.54±4.06	**26.1±12.6**	99±1	100±1	98±2	99±1
Δcop Δcup Δgig	2.55±0.99	4.85±6.21	1.38±0.64	**7.82±3.97**	100±0	99±1	93±5	98±3
Δcop Δcup Δcus	4.47±1.42	6.18±8.05	4.12±2.43	7.34±3.73	100±0	99±1	97±3	99±2
Δcop Δcup Δcus Δgig	3.86±3.22	9.04±9.46	5.92±2.86	10.0±2.00	100±0	99±2	97±1	99±2

[a] The indicated mutant strains of *C. metallidurans* AE104 with or without the additional Δ*gshA* disruption were incubated 20 hours in the presence of Cu(II) provided at the IC_{50} concentration (Table 1) of the respective strain, without copper (negative control) and a live-dead-staining was applied. Cells were incubated for 1 hour at room temperature in saline (live sample) or in 70 % isopropanol (dead sample). The percentage of the dead cells (filter 3 λ_{Ex} 546 nm/λ_{Em} 590 nm) of the total cells (filter 2 λ_{Ex} 450-490 nm/λ_{Em} 520 nm)) is given. Bold-faced values are Q > 1.5 and D > 1, n = 4 wihout copper and n = 6 with copper.

Supplementary Table S3. Mininum and maximum percentage of of dead *C. metallidurans* mutant cells[a].

Bacterial Strain	% dead cells											
	GshA^{+}						Δ*gshA*					
	no Cu			plus Cu			no Cu			plus Cu		
	Min	Median	Max	Min	Median	Max	Min	Median	Max	Min	Median	Max
AE104	1.68	5.29	7.46	**2.72**	**8.01**	**20.2**	1.04	2.47	5.25	**2.97**	**3.62**	**5.62**
Δcop	0.65	3.36	4.20	**3.67**	**9.97**	**18.4**	0.95	1.35	7.29	**2.65**	**7.21**	**23.8**
Δcup	0.00	1.59	6.55	**2.60**	**6.87**	**18.9**	0.77	1.06	2.08	**2.80**	**4.22**	**6.02**
Δcus	0.84	1.89	6.62	**3.28**	**9.91**	**47.6**	0.76	1.19	2.13	**1.96**	**5.46**	**10.5**
Δgig	2.94	3.20	4.47	1.60	5.76	10.0	0.60	2.53	4.49	**1.48**	**4.27**	**6.37**
Δcop Δcup	2.01	3.51	6.82	**3.58**	**9.70**	**17.2**	2.13	13.25	16.1	4.10	9.27	13.3
Δcop Δcus	0.75	4.08	5.38	**11.6**	**19.03**	**52.1**	1.28	2.12	5.49	**6.63**	**12.20**	**21.6**
Δcop Δgig	0.68	1.78	5.79	**2.27**	**7.32**	**29.6**	3.96	4.58	4.88	**15.9**	**25.46**	**38.7**
Δcus Δgig	0.92	1.76	9.20	**5.33**	**8.12**	**72.0**	1.72	2.22	2.80	0.88	3.12	6.94
Δcup Δgig	1.90	4.48	7.27	1.55	2.21	13.0	3.06	4.14	6.05	**8.87**	**10.51**	**13.4**
Δcup Δcus	0.90	4.00	8.51	0.00	1.96	6.50	2.45	3.42	7.45	**4.97**	**19.74**	**37.0**
Δcup Δcus Δgig	3.46	4.14	8.74	0.75	3.48	7.50	0.00	3.65	8.48	0.27	1.62	2.94
Δcop Δcus Δgig	2.46	4.92	9.72	**9.52**	**17.35**	**24.2**	1.35	6.79	11.2	**12.8**	**25.91**	**41.9**
Δcop Δcup Δgig	1.61	2.51	3.54	0.00	1.88	15.2	0.47	1.59	1.88	**1.99**	**7.89**	**13.2**
Δcop Δcup Δcus	2.97	4.51	5.88	0.77	3.37	22.3	0.50	5.18	5.63	**2.14**	**9.02**	**11.4**
Δcop Δcup Δcus Δgig	1.86	2.46	8.65	**1.75**	**6.87**	**27.0**	3.50	5.16	9.87	**6.83**	**10.99**	**11.7**

[a] The indicated mutant strains of *C. metallidurans* AE104 with or without the additional Δ*gshA* disruption were incubated for 20 hours in the presence of Cu(II) provided at the IC_{50} concentration (Table 1) of the respective strain, without copper (negative control) and a live-dead-staining was applied. The minimum, median and maximum values of 4 experiments (no copper) or 6 experiments (plus copper) is provided. Bold-faced results indicate strains with the respective Min, Median and Max values were always higher in the presence of copper than without.

Supplementary Table S4. Metal content of *C. metallidurans* mutant strains

Bacterial strain	10^3 Cu/cell	10^3 Zn/cell	10^3 Fe/cell	10^6 Mg/cell	Cu(II), (µM)	10^3 Cu/cell	10^3 Zn/cell	10^3 Fe/cell	10^6 Mg/cell	Bacterial strain
AE104	5.97±1.00	55.6±7.7	595±44	8.21±0.70	25	32.8±5.6	43.1±7.4	477±142	8.06±1.04	AE104
AE104 ΔgshA	6.11±1.41	42.6±1.2	**377±41**	7.87±0.75	25	23.0±2.5	46.4±14.9	434±203	7.58±0.14	AE104 ΔgshA
AE104 ΔgshA (makerless)	5.19±0.90	47.3±16.1	513±37	7.97±1.41	25	23.4±0.7	44.2±9.2	411±112	7.60±0.81	AE104 ΔgshA (real)
Δcop ΔgshA	4.62±0.67	43.1±4.6	460±79	7.02±0.29	25	**125±25**	42.6±11.1	**337±85**	7.25±0.43	*Δcop ΔgshA*
Δcup ΔgshA	5.50±0.94	39.7±7.2	**341±53**	6.87±0.82	1	19.1±2.6	38.5±9.2	**280±34**	6.63±0.64	*Δcup ΔgshA*
Δcus ΔgshA	5.13±0.15	48.1±4.5	450±42	8.11±0.68	25	36.3±5.7	45.8±13.0	410±109	8.50±1.36	*Δcus ΔgshA*
Δgig ΔgshA	5.02±0.47	49.7±12.7	441±56	7.70±0.61	25	24.4±3.5	45.2±9.3	372±86	7.69±0.36	*Δgig ΔgshA*
Δcop Δcup ΔgshA	6.03±0.58	46.2±4.8	524±23	9.71±0.20	0.02	13.6±4.0	43.4±5.0	409±65	8.83±0.94	*Δcop Δcup ΔgshA*
Δcop Δcus ΔgshA	5.00±0.53	40.8±7.1	**392±51**	7.76±0.46	1	**45.5±20.3**	37.5±5.3	383±11	8.85±2.69	*Δcop Δcus ΔgshA*
Δcop Δgig ΔgshA	6.69±0.41	55.4±10.0	489±39	8.98±0.82	25	**253±17**	39.9±9.1	**343±84**	7.78±0.26	*Δcop Δgig ΔgshA*
Δgig Δcus ΔgshA	6.58±0.74	47.4±10.1	396±75	7.28±0.31	25	25.3±1.3	40.2±12.8	**315±90**	7.50±0.44	*Δgig Δcus ΔgshA*
Δcup Δgig ΔgshA	6.02±0.75	43.7±3.7	441±87	7.94±0.62	1	17.5±0.8	39.8±6.8	**324±2**	7.35±0.20	*Δcup Δgig ΔgshA*
Δcup Δcus ΔgshA	5.09±0.39	40.2±4.9	428±82	7.13±0.16	1	19.6±1.1	37.7±3.7	**338±58**	7.60±0.92	*Δcup Δcus ΔgshA*
Δcup Δcus Δgig ΔgshA	**3.30±1.03**	**24.8±8.2**	**313±22**	**14.7±2.0**	0.02	8.0±1.3	28.8±3.9	**300±70**	**16.2±2.5**	*Δcup Δcus Δgig ΔgshA*
Δcop Δcus Δgig ΔgshA	4.71±0.62	**34.5±5.2**	**314±50**	**18.5±0.8**	1	33.3±12.4	33.4±3.2	**286±7**	**25.8±0.4**	*Δcop Δcus Δgig ΔgshA*
Δcop Δcup Δgig ΔgshA	**3.68±0.67**	**33.0±8.7**	**338±44**	**20.5±1.9**	0.02	11.5±1.2	37.8±5.4	306±81	**22.7±3.5**	*Δcop Δcup Δgig ΔgshA*
Δcop Δcup Δcus ΔgshA	5.64±0.44	43.4±5.8	407±68	7.60±0.83	0.02	9.8±0.8	41.9±13.7	311±89	7.63±0.81	*Δcop Δcup Δcus ΔgshA*
Δcop Δcup Δcus Δgig ΔgshA	5.78±1.10	51.0±10.9	**351±87**	**22.4±3.7**	0.02	12.0±1.4	37.1±9.1	**273±30**	**20.2±2.2**	*Δcop Δcup Δcus Δgig ΔgshA*
AE104	6.88±1.75	40.1±10.9	486±77	8.37±1.46	25	33.6±6.8	38.3±6.3	536±106	8.15±0.64	AE104
AE104 *ΔgshA*	6.88±1.29	36.2±5.3	**267±26**	7.17±0.65	1	**20.5±3.9**	42.2±5.8	516±27	9.02±0.34	AE104
AE104 *ΔgshA* (markerless)	6.92±2.10	37.7±4.8	363±60	6.86±0.53	0.02	**10.8±0.8**	42.2±9.1	451±9	8.14±0.27	AE104
Δcop	7.83±3.20	42.0±7.7	429±69	7.68±0.69	25	**52.4±8.4**	33.5±4.4	522±27	7.54±0.39	*Δcop*
Δcup	9.00±3.41	35.2±8.0	452±104	7.50±0.60	1	19.9±6.1	34.8±6.9	429±41	7.26±0.40	*Δcup*
Δcus	7.59±2.64	40.9±7.6	386±84	7.92±1.20	25	49.6±12.9	30.2±2.8	366±14	7.61±0.73	*Δcus*
Δgig	7.01±1.21	39.9±8.9	418±51	7.47±0.45	25	26.2±2.7	29.4±2.4	423±65	7.13±0.33	*Δgig*
Δcop Δcup	6.48±2.23	27.2±7.1	398±91	**12.8±2.9**	0.02	8.0±1.0	30.8±7.0	359±24	**16.7±1.7**	*Δcop Δcup*
Δcop Δcus	7.06±1.33	37.9±4.6	443±59	7.69±0.75	1	26.0±3.2	30.1±2.9	466±35	7.04±0.45	*Δcop Δcus*
Δcop Δgig	5.67±1.05	31.9±6.9	436±67	8.10±1.30	25	36.3±7.5	35.7±3.5	481±28	8.16±0.25	*Δcop Δgig*
Δgig Δcus	7.33±2.02	38.4±5.1	421±77	7.23±1.27	25	28.1±3.0	28.7±1.9	430±33	7.33±0.31	*Δgig Δcus*
Δcup Δgig	6.59±2.29	34.8±7.0	392±55	7.32±0.79	1	20.6±7.0	33.7±9.1	436±17	7.52±0.50	*Δcup Δgig*
Δcup Δcus	5.22±1.16	27.1±5.9	367±45	6.56±1.08	1	20.3±3.5	35.5±7.4	453±24	7.49±0.25	*Δcup Δcus*
Δcup Δcus Δgig	6.11±2.12	32.2±9.6	425±141	**15.3±2.5**	0.02	9.7±0.3	32.3±4.3	385±28	**15.3±0.4**	*Δcup Δcus Δgig*
Δcop Δcus Δgig	6.71±2.21	34.5±6.5	397±59	**12.6±2.3**	1	30.6±8.4	32.5±5.1	444±26	**17.4±0.6**	*Δcop Δcus Δgig*
Δcop Δcup Δgig	5.85±1.03	33.0±5.3	368±34	**14.7±1.8**	0.02	7.4±2.6	31.2±9.7	363±38	**17.2±1.3**	*Δcop Δcup Δgig*
Δcop Δcup Δcus	8.04±3.82	31.6±6.7	394±80	**17.4±2.6**	0.02	8.3±3.2	31.8±5.6	378±21	**19.7±1.8**	*Δcop Δcup Δcus*
Δcop Δcup Δcus Δgig	6.32±1.88	33.6±9.8	387±122	**19.0±6.5**	0.02	9.2±0.3	34.8±3.9	369±15	**19.6±0.7**	*Δcop Δcup Δcus Δgig*

Bold: (Q ≥ 1.5 OR Q ≤ 0.66) AND D>1. Metal content of *C. metallidurans* cells, measured by ICP-MS. Cells were incubated without (negative control) or with copper at indicated concentrations. Cells were mineralized in 70 % nitric acid at 70 °C for 2 hours and samples were diluted to a final concentration of 2 % nitric acid. Grey field, parent strain AE104 with different copper concentrations.

Supplementary Table S5. Bacterial strains

Bacterial strain	Description/genotype/plasmid	Reference
C. metallidurans		
AE104	Plasmid-free derivative of CH34	(1)
DN861	AE104 Δ*gshA*	this study
DN656	AE104 Δ*cupCAR*	(2)
DN657	AE104 Δ*gigPABT*	(2)
DN686	AE104 Δ$copA_2B_2C_2D_2$	(2)
DN729	AE104 Δ*cusDCBAF*	this study
DN845	AE104 Δ*cop* Δ*cup*	this study
DN846	AE104 Δ*cop* Δ*cus*	this study
DN847	AE104 Δ*cop* Δ*gig*	this study
DN848	AE104 Δ*gig* Δ*cus*	this study
DN849	AE104 Δ*cup* Δ*gig*	this study
DN850	AE104 Δ*cup* Δ*cus*	this study
DN851	AE104 Δ*gig* Δ*cus* Δ*cup*	this study
DN852	AE104 Δgig Δ*cus* Δ*cop*	this study
DN853	AE104 Δ*gig* Δ*cup* Δ*cop*	this study
DN854	AE104 Δ*cop* Δ*cup* Δ*cus*	this study
DN855	AE104 Δ*cop* Δ*cup* Δ*cus* Δ*gig*	this study
E. coli (used for conjugation)		
ECA962	pECD1386, used for *cusF*-*lacZ* fusion	this study
ECB249	pECD1667, used for *gigT*-*lacZ* fusion	this study
ECB250	pECD1668, used for *gshA* disruption	this study

Supplementary Table S6. Primers

**Name and purpose**	**Sequence (5'→3')**	**Reference**
Deletion of *gshA*		
RmgshAcreloxBglII	AAAAGATCTTGCCTGCGGGGCATAGAG	this study
delta gshA-RM OR	CAGCAATTGGATGAGATGCGGGACCATATCCAT	this study
Rm2 DgshA ApaI fwd	CTCGGGCCCACCGACCCGAATCCGATCCTC	this study
Rm2 DgshA SacI rev	ATGGAGCTCGGCGGATCCTTGCGCATCA	this study
RT-PCR		
NW Rmet_ 4683 ApaI	AAAGGGCCCAATTTCCCGACGGTCCTGCGGC	this study
NW Rmet_4681 ApaI	AAAGGGCCCGCCTAGATCTGGCCGATATCC	this study
NW Rmet_4685 MunI	AAACAATTGGAAGTGCTACGGCGTGGCCA	this study
NW doxX MunI	AAACAATTGAGCAGCCCGGTGACCTTGGT	this study
Disruption of *gshA*		
gshA Rm dis 586 Pst	AAACTGCAGTACCTGCTGCCGCCGTTG	this study
gshA Rm dis 901 Xba	AAATCTAGAGCCCTTCACCTCGGACGG	this study
Deletion of *cusDCBAF*		
NW cus-like MunI	AAACAATTGGTGGATATCGGATCCAGAAC	this study
NW cus-like NotI	AAAGCGGCCGCGATCTACGAGGATGTCAAAAC	this study
NW cus-like ApaI	AAAGGGCCCCGCGATCGAGCCGCGCCGAC	this study
NW cus-like AgeI	AAAACCGGTAGTGGGCCATCGCAATCGAAGGG	this study

Supplementary Figure S1. Maps of genetic determinants encoding copper resistance. Maps are shown of determinants in the indicated regions with NPKM values (nucleotide activities per kilobase of exon model per million mapped reads) on one DNA strand (red) or the other direction of transcription (blue). Values in orange and light blue are transcripts resulting from multiple homologous DNA regions, which could not be associated to a single locus. Above are the Rmet locus and gene names, the mean NPKM and response values (3). TSSs (flags) are indicated with the corresponding TSS score (not: RpoD score), white for scores < 50 with no promoter consensus motifs indicated, red shades for strong (>1000, red), medium (100-1000, orange) or weak (50-100, light orange) RpoD promoters, blue shades (strong, medium, light) according if not associated to the RpoD model. The TSS determination and association of the TSS to RpoD are published (4)]. The maps already published (4) of *cop2, cus,* and *cup* are also given. The black bars indicate the position of the deletions in the mutants used in this study.

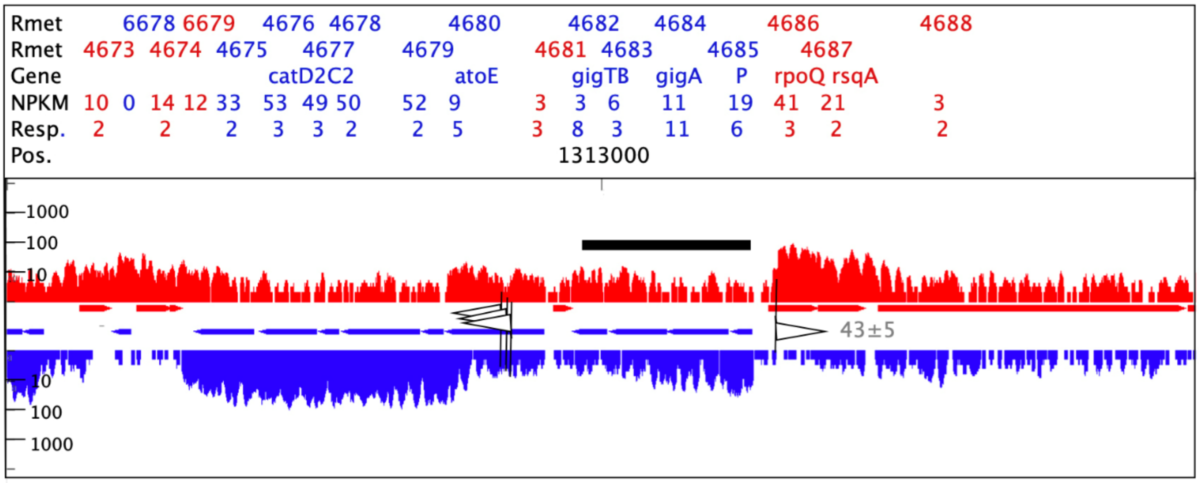

Panel A. The *gig* determinant on the chromid. The *gigAp* promoter has not yet been identified.

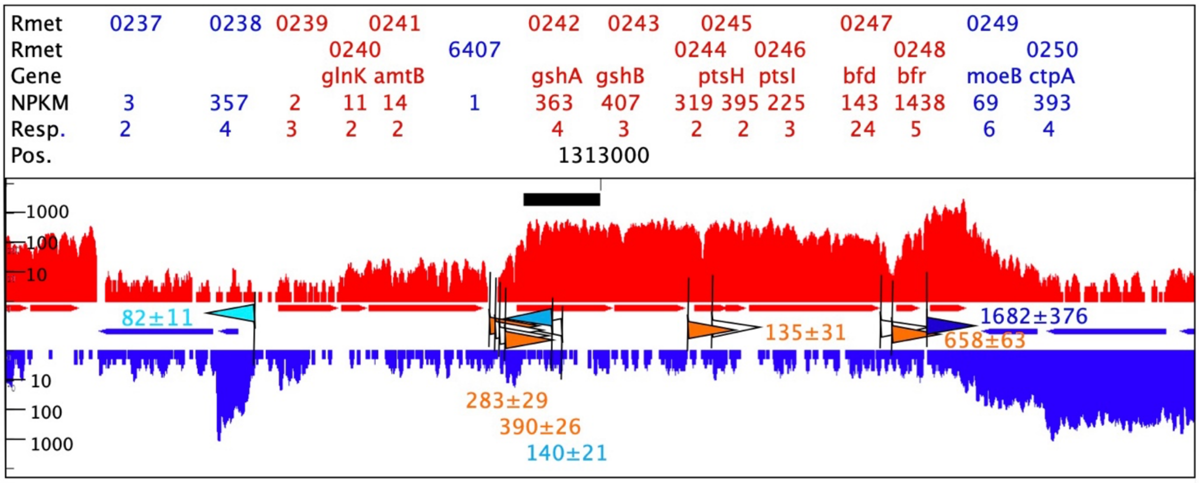

Panel B. The *gshAB* region on the chromosome. The position of the marker-free deletion is indicated as a black bar. Most mutants described in this publication contain an interruption of *gshA* constructed by integration of a plasmid.

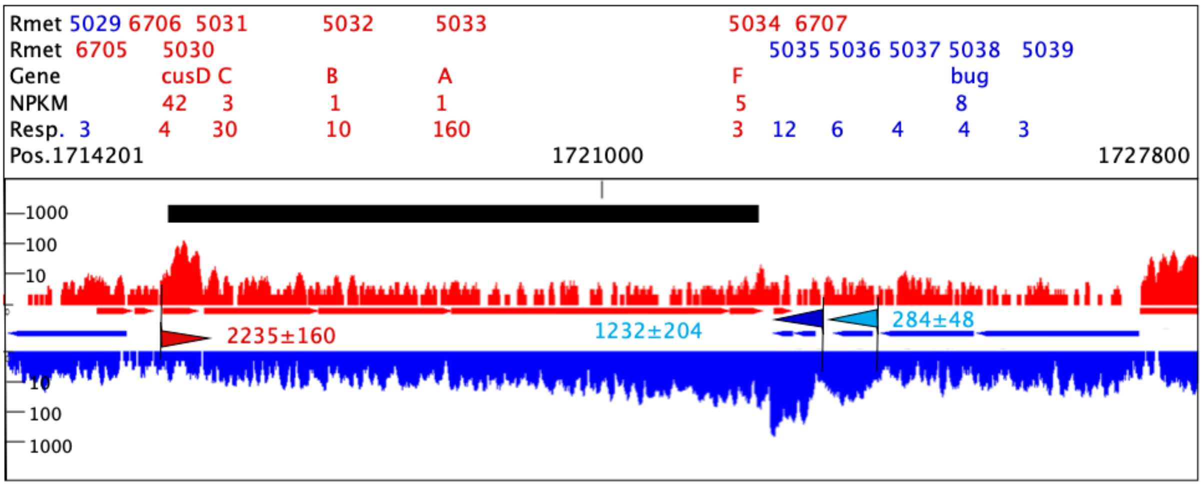

Panel C. The *cus* determinant on the chromid.

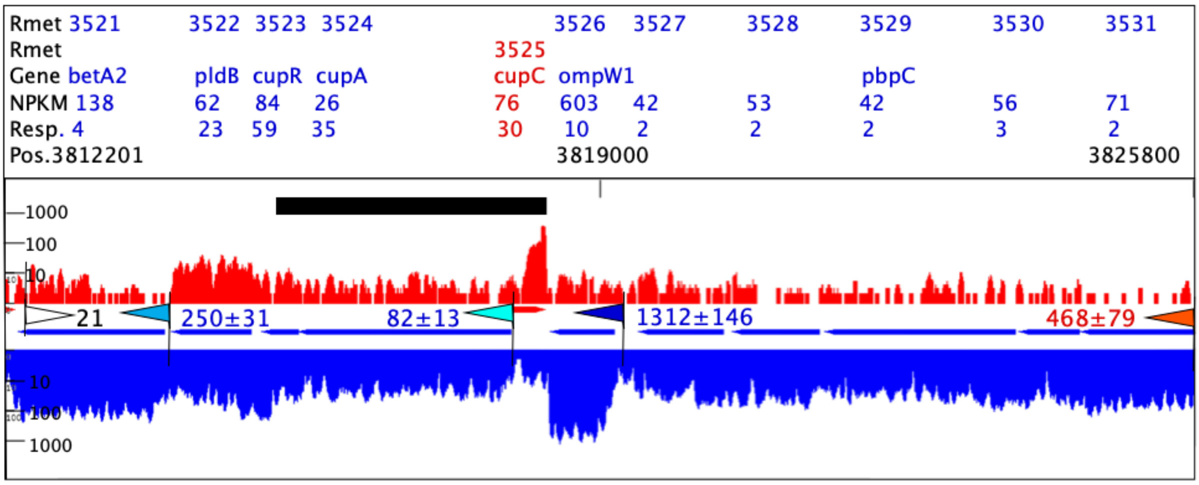

Panel D. The *cup* determinant on the chromosome.

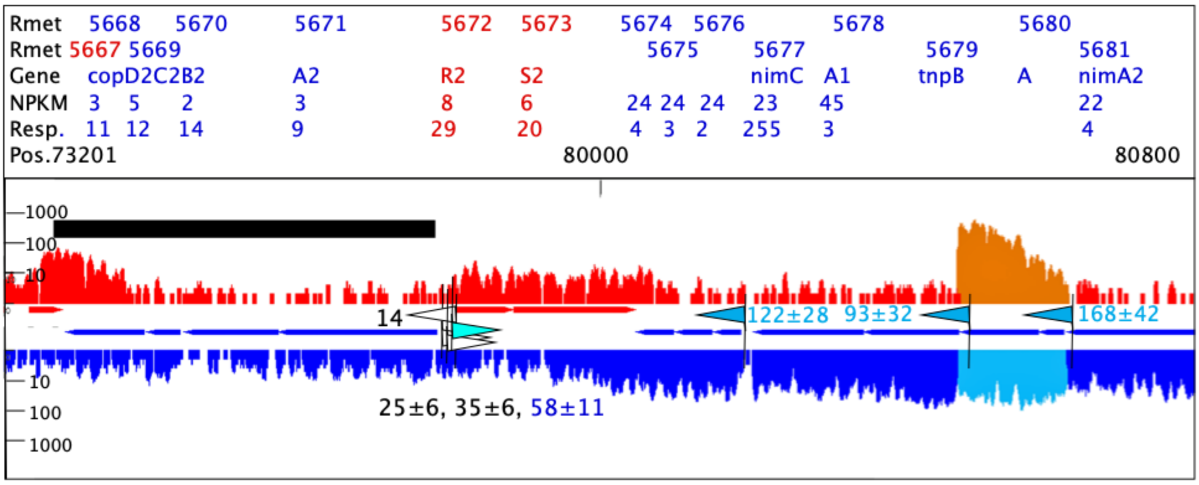

Panel E. The *cop2* determinant on the chromid

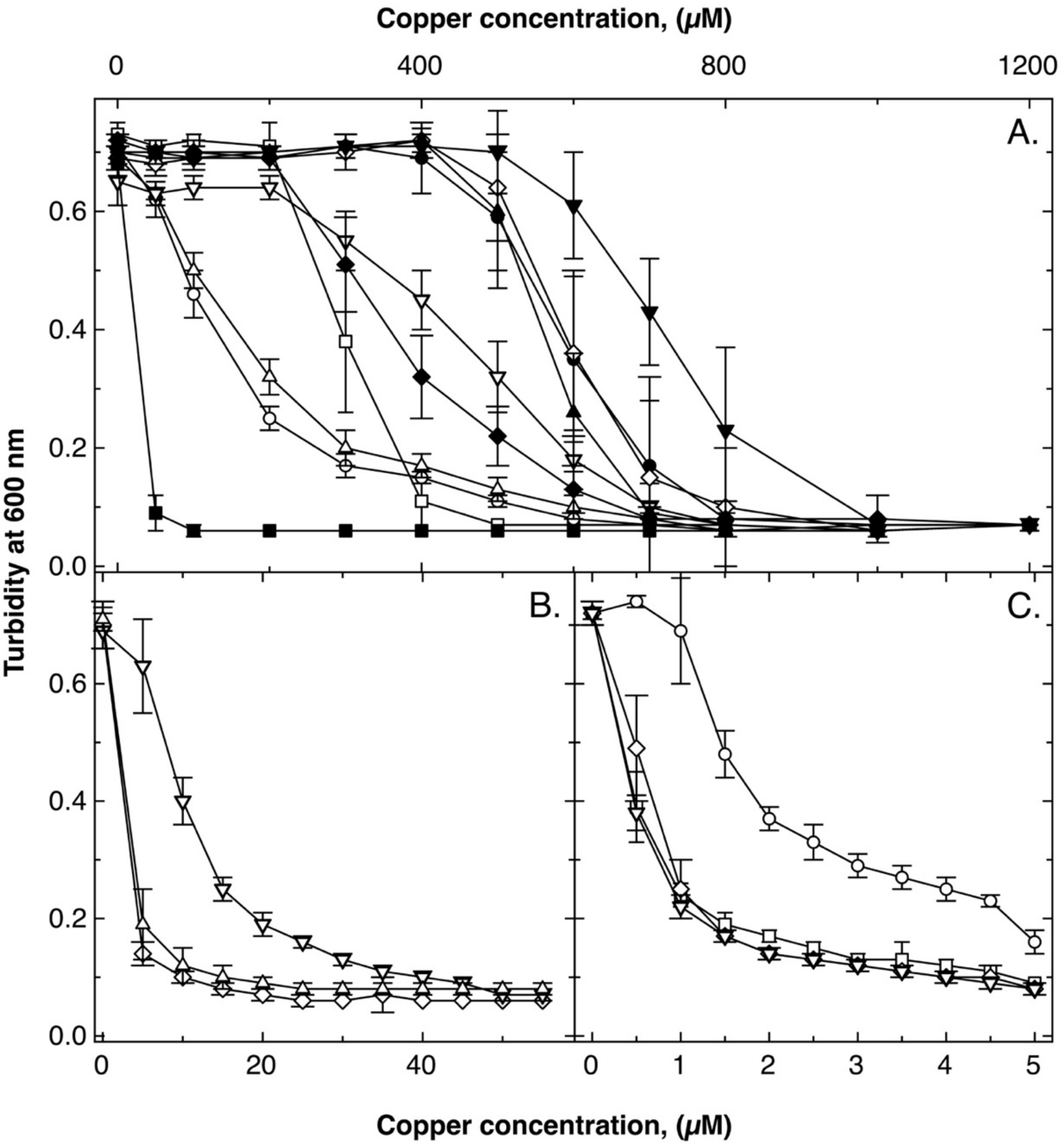

Supplementary Figure S2. Copper resistance of deletion mutants. Single, double, triple and quadruple deletion mutants were characterized in dose-response experiments at various copper concentrations from high (Panel A) via medium (Panel B) to low (Panel C)**.** Parent, single, double and one triple mutant in Panel A: AE104 parent (closed circles, ●), Δ*cup* (closed squares, ■), Δ*cop* (closed diamonds, ◆), Δ*cus* (closed inverted triangles, ▼), Δ*gig* (closed trianges, ▲), Δ*gshA* (open squares, □), Δ*cop* Δ*cus* (open triangles, △), Δ*cop* Δ*gig* (open inverted triangles, ▽), Δ*gig* Δ*cus* (open diamonds, ◇), and Δ*cop* Δ*cus* Δ*gig* (open circles, ○). Panel B shows the double mutants Δ*cup* Δ*cop* (opend diamonds, ◇*)*, Δ*cup* Δ*gig* (open inverted triangles, ▽*)*, Δ*cup* Δ*cus* (open triangles, △), and Panel C the triple mutants Δ*cop* Δ*cup* Δ*cus* (opend diamonds, ◇*)*, Δ*cup* Δ*cop* Δ*gig* (open squares, □*)*, Δ*cup* Δ*cus* Δ*gig* (open circles, ○) and the quadruple mutant Δ*cop* Δ*cup* Δ*cus* Δ*gig* (open inverted triangles, ▽). Please note the different scales on the x-axises, n ≥ 3, deviations shown.

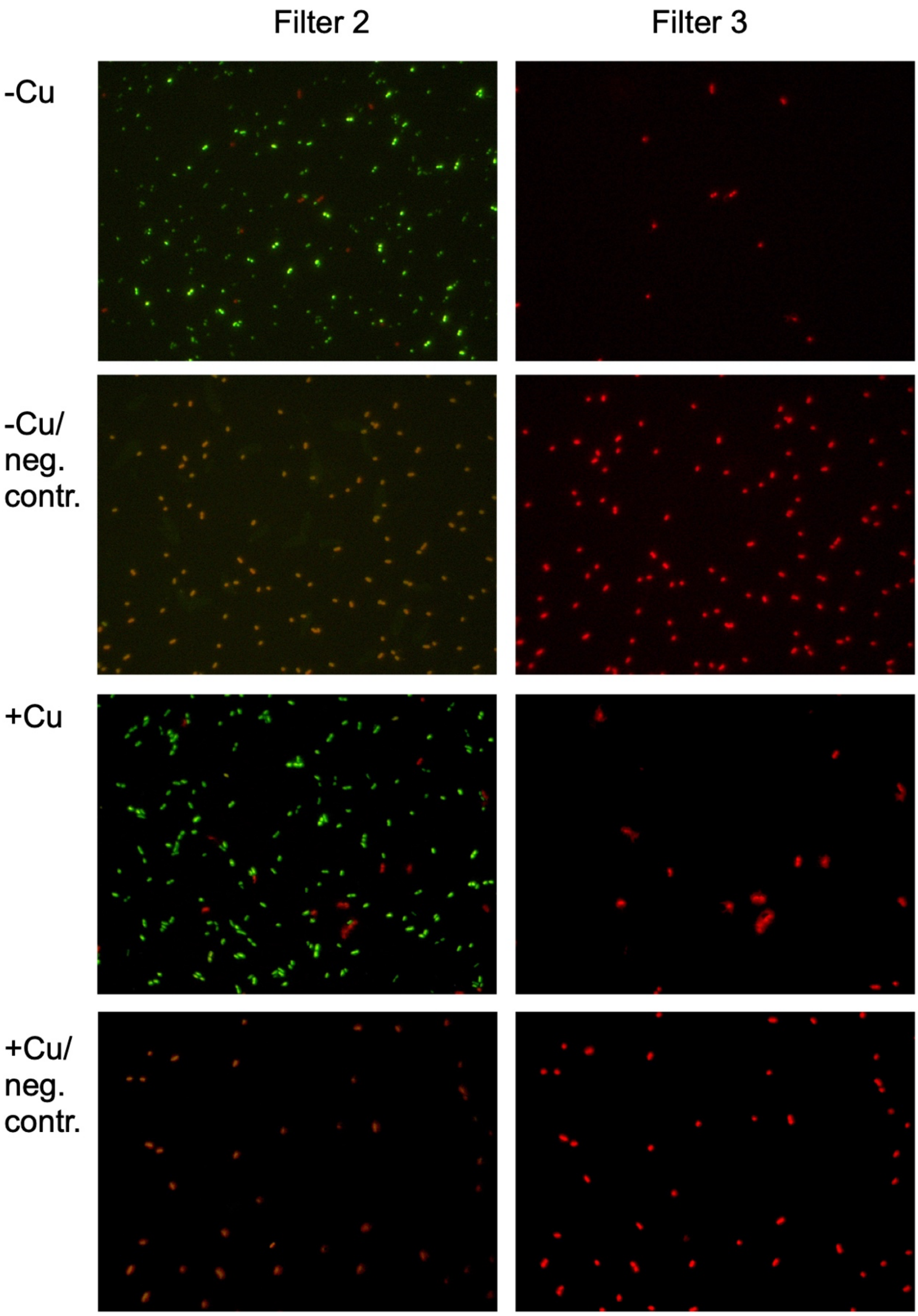

Supplementary Figure S3. Live and dead cells in cultures of *C. metallidurans* AE104. Cells were incubated für 20 hours in the presence of copper at IC_{50} concentrations of the respective strain (Table 1) or without copper (negative control) and a live-dead-staining was applied. Filter 3 (λ_{Ex} 546 nm/λ_{Em} 590 nm) was used to determine dead cells and filter 2 (λ_{Ex} 450-490 nm/λ_{Em} 520 nm) to determine total cells. When using filter 2 living cells fluoresce green and dead cells red. While using filter 3 living cells do not fluoresce and dead cells fluoresce red.

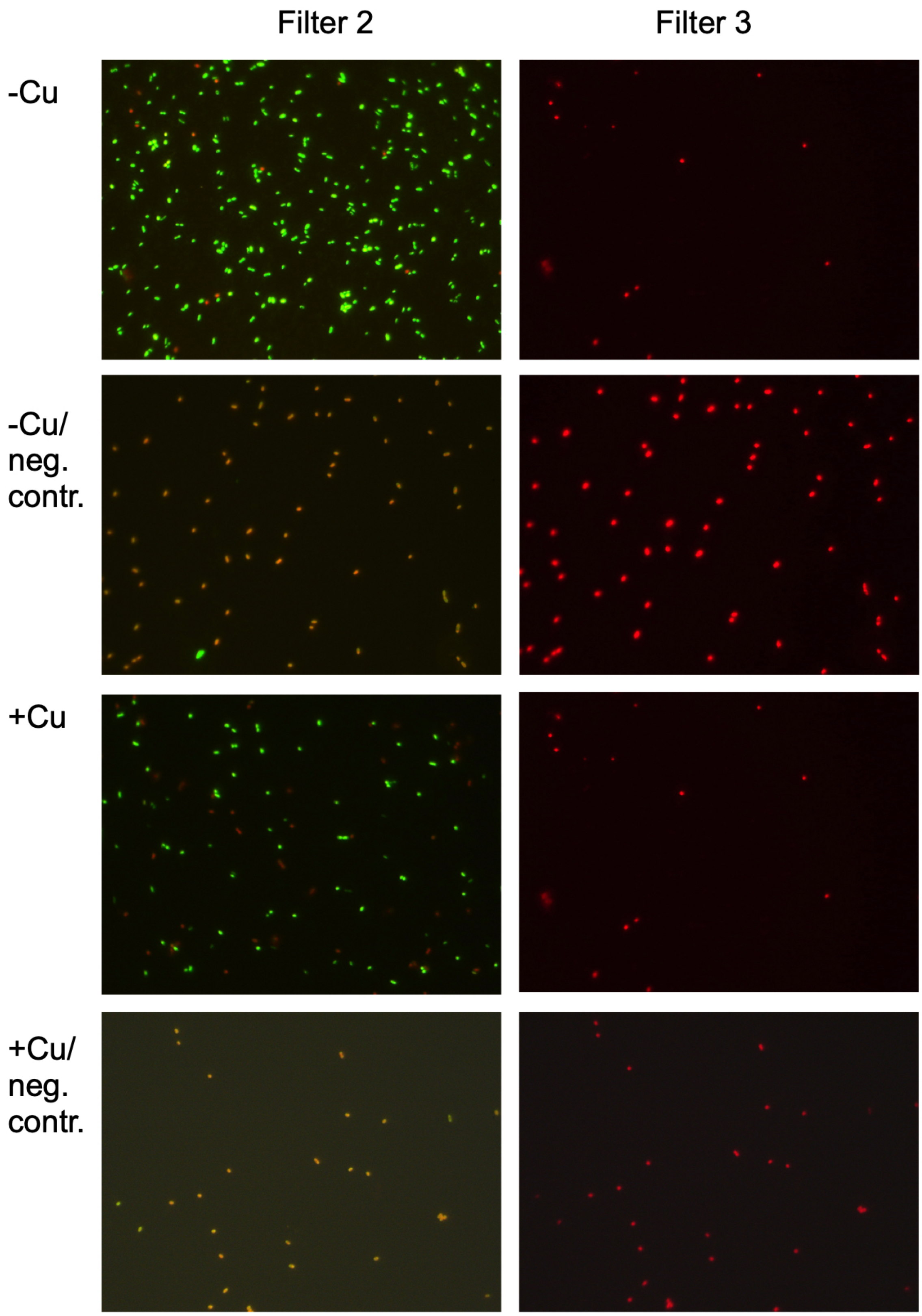

Supplementary Figure S4. Determination of dead cells in cultures of *C. metallidurans* mutant AE104 ∆*cop* ∆*gig* ∆*gshA*. The strain was incubated for 20 hours with copper at its respective IC_{50} concentration (table 1) or without copper (negative control) and a live-dead-staining was applied. Filter 2 (λ_{Ex} 450-490 nm/λ_{Em} 520 nm) was used to determine total cells and filter 3 (λ_{Ex} 546 nm/λ_{Em} 590 nm) was used to determine dead cells. When using filter 2 living cells fluoresce green and dead cells red. While using filter 3 living cells do not fluoresce and dead cells fluoresce red.

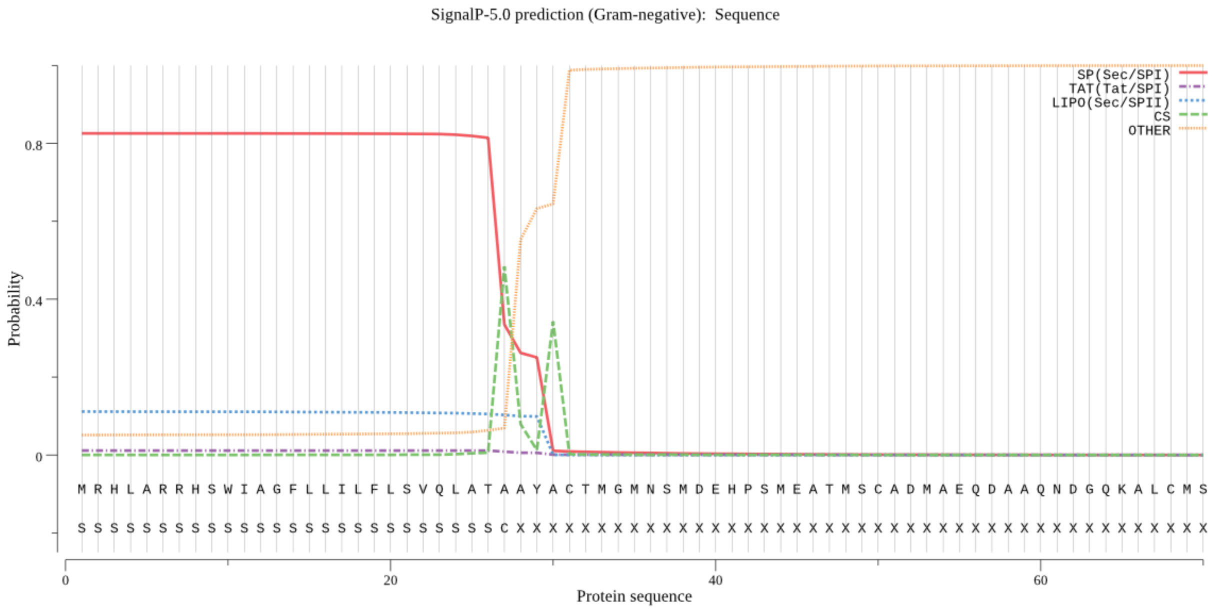

Supplementary Figure S5. SignalP prediction of the CusD leader. The conserved double arginine motif is clearly seen, as is a conserved cysteine for attachment of a lipid acid (5).

Literature cited in the supplement

1. Mergeay M, Nies D, Schlegel HG, Gerits J, Charles P, van Gijsegem F. 1985. *Alcaligenes eutrophus* CH34 is a facultative chemolithotroph with plasmid-bound resistance to heavy metals. J Bacteriol 162:328-334.
2. Wiesemann N, Mohr J, Grosse C, Herzberg M, Hause G, Reith F, Nies DH. 2013. Influence of copper resistance determinants on gold transformation by *Cupriavidus metallidurans* strain CH34. J Bacteriol 195:2298-2308.
3. Große C, Kohl T, Herzberg M, Nies DH. 2022. Loss of mobile genomic islands in metal resistant, hydrogen-oxidizing *Cupriavidus metallidurans*. Appl Environ Microbiol 88:e02048-21.
4. Große C, Grau J, Große I, Nies DH. 2022. Importance of RpoD- and non-RpoD-dependent expression of horizontally acquired genes in *Cupriavidus metallidurans*. Microbiol Spectr doi: 10.1128/spectrum.00121-22: 10.1128/spectrum.00121-22.
5. Almagro Armenteros JJ, Tsirigos KD, Sønderby CK, Petersen TN, Winther O, Brunak S, von Heijne G, Nielsen H. 2019. SignalP 5.0 improves signal peptide predictions using deep neural networks. Nat Biotechnol 37:420-423.
